# Quantum-well metasurface for free-space-accessible enhanced nonlinear polarization

**DOI:** 10.1038/s41565-026-02268-0

**Published:** 2026-09-02

**Authors:** Pernille Undrum Fathi, Irene Occhiodori, Patrick Devaney, Amberly Ricks, Rithvik Ramesh, Yiwei Ju, Moaz Waqar, Theodore P. Letsou, Christina M. Spägele, Hyunseung Jung, Igal Brener, Xiaoqing Pan, Marcus Ossiander, Seth R. Bank, Federico Capasso

**Affiliations:** 1 Harvard John A. Paulson School of Engineering and Applied Sciences, Harvard University, Cambridge, MA USA; 2 Department of Materials, ETH Zürich, Zurich, Switzerland; 3 Microelectronics Research Center and Electrical and Computer Engineering Department, University of Texas at Austin, Austin, TX USA; 4 Department of Electrical Engineering, California Institute of Technology, Pasadena, CA USA; 5 Department of Materials Science and Engineering, University of California, Irvine, Irvine, CA USA; 6 Department of Electrical Engineering and Computer Science, Massachusetts Institute of Technology, Cambridge, MA USA; 7 Center for Integrated Nanotechnologies, Sandia National Laboratories, Albuquerque, NM USA; 8 Department of Physics and Astronomy, University of California, Irvine, Irvine, CA USA; 9 Irvine Materials Research Institute (IMRI), University of California, Irvine, Irvine, CA USA; 10 Institute of Experimental Physics, Graz University of Technology, Graz, Austria

**Keywords:** Nonlinear optics, Nonlinear optics, Nanophotonics and plasmonics, Optical physics

## Abstract

Nonlinear frequency conversion underpins important technologies such as telecommunications and quantum computation; however, weak nonlinearities and architectures that resist miniaturization currently limit devices’ efficiency and widespread adoption. Here we combine a band-structure-engineered GaAs/AlGaAs multi-quantum-well heterostructure with a high-quality-factor dielectric metasurface and symmetry-broken guided-mode field profiles to enhance the material nonlinear susceptibility. By engineering a resonant interband transition in the heterostructure, we realize a second-order nonlinear tensor element of 1.6 nm V^−1^ at 1.57 μm wavelength. We then make it free-space accessible and boost the effective nonlinearity to ~14 nm V^−1^ using a metasurface patterned on the material. Our proof-of-concept experiment establishes that combining interband-transition engineering and metasurfaces enables giant effective nonlinearities in the near-infrared to visible spectrum. This addresses material and device-level constraints in nonlinear photonics, providing a scalable route to compact, efficient devices.

## Abstract (web summary)

Interband-engineered quantum wells integrated with a resonant metasurface yield giant near-infrared-to-visible optical nonlinearities.

## Main

Nonlinear optical processes are fundamental to modern photonics, enabling frequency mixing^1^, ultra-fast signal processing^2^, broadband and pulsed light sources^3–5^, high-speed modulation^6^ and the generation of entangled photon pairs for quantum communication^7–9^. Efficient nonlinear devices require light with high electric field strengths and materials with large nonlinear susceptibilities. Conventional nonlinear devices rely on a handful of bulk crystals with weak intrinsic nonlinearities, compensating at the device level through long coherent propagation distances^10,11^. This hinders device miniaturization and requires phase-matched propagation conditions, which often necessitates complex dispersion engineering.

Metasurfaces can enhance incident light fields and thereby enable efficient nonlinear devices with short propagation distances^12–21^. While these devices can compensate for low nonlinearity through high quality factors, this introduces a trade-off with the operational bandwidth and practical fabrication constraints. Still, the nonlinear susceptibilities of the underlying material platforms fundamentally limit their performance.

Semiconductor multi-quantum-well (MQW) heterostructures can overcome the limited nonlinear response of bulk crystals. Band-structure engineering can create MQW states and control the expectation value of the electron position in each state. Intersubband transitions between states with large spatial offsets therefore produce strong electron displacement, which can resonantly enhance the nonlinear susceptibilities close to the intersubband transition energy^22,23^. However, in III–V semiconductors, intersubband transition energies are limited by the heterostructure band offset, restricting operating wavelengths to the mid-infrared, where this approach to tailored nonlinear response has been used to demonstrate second-order susceptibilities that exceed bulk-crystal values by orders of magnitude^24–26^. Field enhancement in plasmonic and Mie resonators can improve mid-infrared nonlinear conversion efficiencies^12,27,28^. Wide-band-offset materials can push intersubband engineering towards near-infrared wavelengths^29^, but demonstrated nonlinear heterostructures have been restricted to materials with loss at the pump and the harmonic wavelengths^30,31^.

**Fig. 1:**
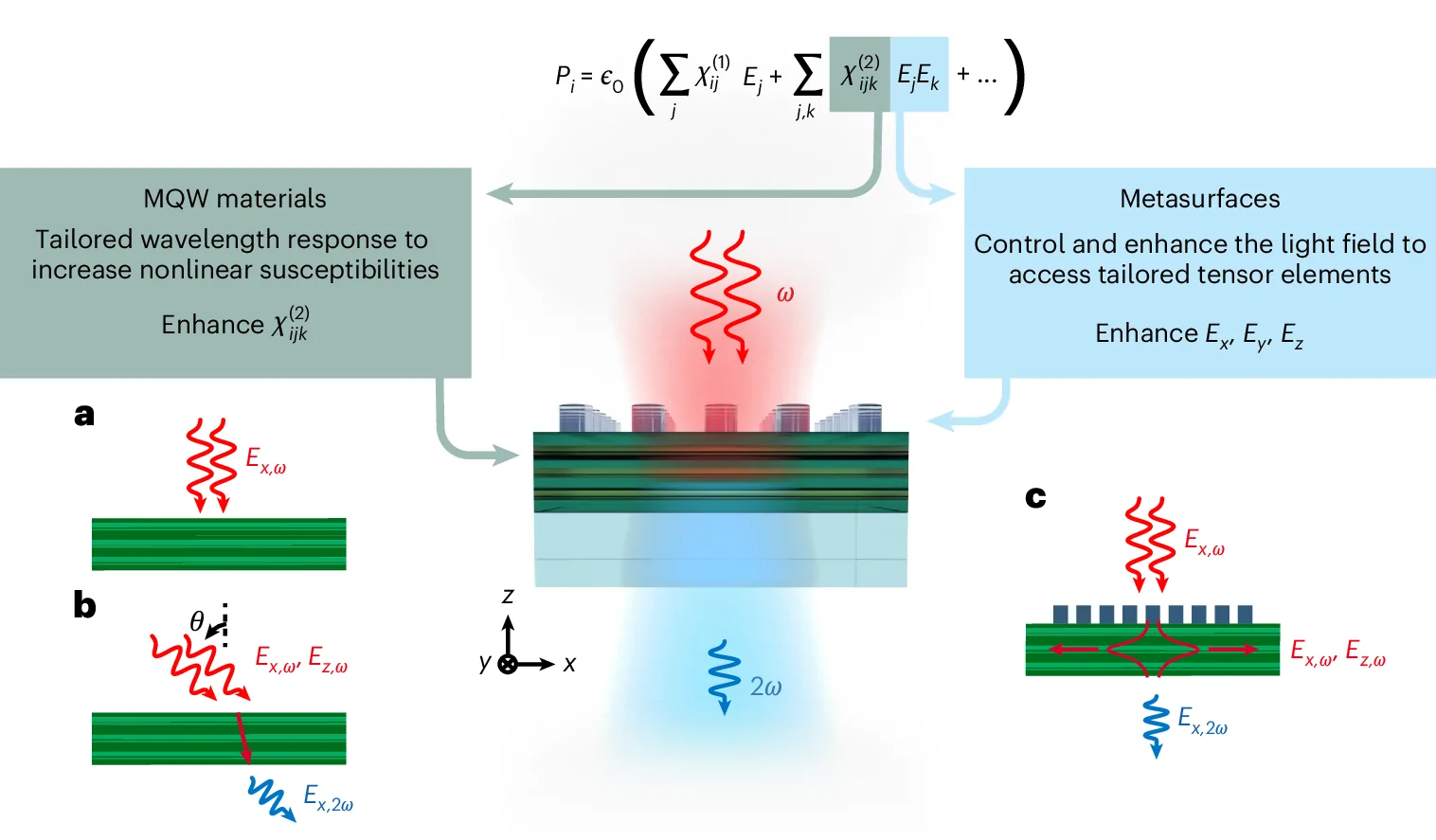
Tailoring all components of the second-order polarization. Combining asymmetric MQWs, which enable resonance enhancement of higher-order susceptibilities, with metasurfaces, which enable precise control and enhancement of electromagnetic fields, enables devices in which every factor of a nonlinear polarization term is tailored to the application. Here we focus on the second-order term. *P* denotes the polarization induced by the incident light fields *E*, which is scaled by the vacuum permittivity *ε*_0_, the linear susceptibility *χ*^(1)^ and the second-order nonlinear *χ*^(2)^ susceptibility. **a**, At normal incidence, selection rules prohibit second-harmonic generation enhanced by MQW-interband transitions. **b**, At oblique incidence (at angle *θ*), selection rules are fulfilled, and second-harmonic generation is possible. For free-space excitation, the conversion efficiency is limited by the maximum propagation angle achievable within the high-index medium. Note that this material requires two different polarizations for the pump photons. **c**, Metasurfaces enable polarization conversion and resonant field enhancement, thereby enabling enhanced second-harmonic generation.

An alternative route is to engineer transitions between MQW states located in different bands, that is, interband transitions, which we refer to here as MQW-interband transitions to distinguish them from their bulk-crystal counterparts^32,33^. Recent work predicted^34^ and experimentally verified^35^ a MQW-interband-transition-engineered GaAs/AlGaAs heterostructure with a second-order nonlinear susceptibility *χ*^(2)^ that exceeds that of bulk GaAs by an order of magnitude. For second-harmonic generation, the nonlinear response *P*_*i*_(2*ω*) along a direction *i* in response to given incident light field strengths *E*_*j*_(*ω*) and *E*_*k*_(*ω*) polarized along the *j* and *k* directions, respectively, with light frequency *ω*, scales as ${P}_{i}(2\omega )\propto {\chi }_{ijk}^{(2)}{E}_{j}(\omega ){E}_{k}(\omega )$. Maximizing this response therefore requires a large second-order nonlinear susceptibility tensor element ${\chi }_{ijk}^{(2)}$, and large, comparable electric pump field strengths *E*_*j*_ ≈ *E*_*k*_. While the MQWs provide one of the largest second-order nonlinear tensor elements in this spectral region, they do so using the tensor elements ${\chi }_{xzx}^{(2)},{\chi }_{xxz}^{(2)},{\chi }_{yzy}^{(2)},{\chi }_{yyz}^{(2)}$, which require one in-plane and one out-of plane pump field component. This poses a challenge for free-space excitation (Fig. 1a): normal incident light cannot directly provide the required *E*_*z*_ field, while facet illumination provides the required field components but limits the interaction area via the film thickness. Oblique incidence can generate an out-of-plane field component (Fig. 1b), but the high refractive index of III–V semiconductors reduces the free-space-accessible internal propagation angle, keeping the out-of-plane field magnitude small.

Here, to address this issue, we combine designed MQW-interband transitions with dielectric metasurface field enhancement to realize giant, free-space-accessible effective nonlinearities for near-infrared-to-visible light conversion. We use the metasurface to excite a guided-mode resonance (GMR) to convert the incident polarization and enhance the local field product driving second-harmonic generation. This co-design approach simultaneously enhances the key factors contributing to the second-order nonlinear polarization product and yields an effective, free-space-accessible nonlinear tensor element that behaves like ${\chi }_{xxx}^{(2)}$ of a bulk crystal (the indices *xxx* denote that *x*-polarized incident light fields generate *x*-polarized second-harmonic light). We demonstrate an effective second-order nonlinear susceptibility magnitude at visible and near-infrared frequencies of ${\chi }_{{\rm{eff}},{\rm{MQW}}+{\rm{MS}}}^{(2)}={\chi }_{xxx,{\rm{MQW}}+{\rm{MS}}}^{(2)}\approx 14$  nm V^−1^ for the co-designed (MQW + metasurface) sample.

## Material design and characterization

Inversion-symmetric materials cannot generate second-harmonic radiation because the material polarization induced by the positive and negative half-cycles of incoming light cannot differ. Conversely, a strong spatial asymmetry, that is, an environment that favours electron motion in one but not the opposite direction, is a prerequisite for a large second-order susceptibility.

**Fig. 2:**
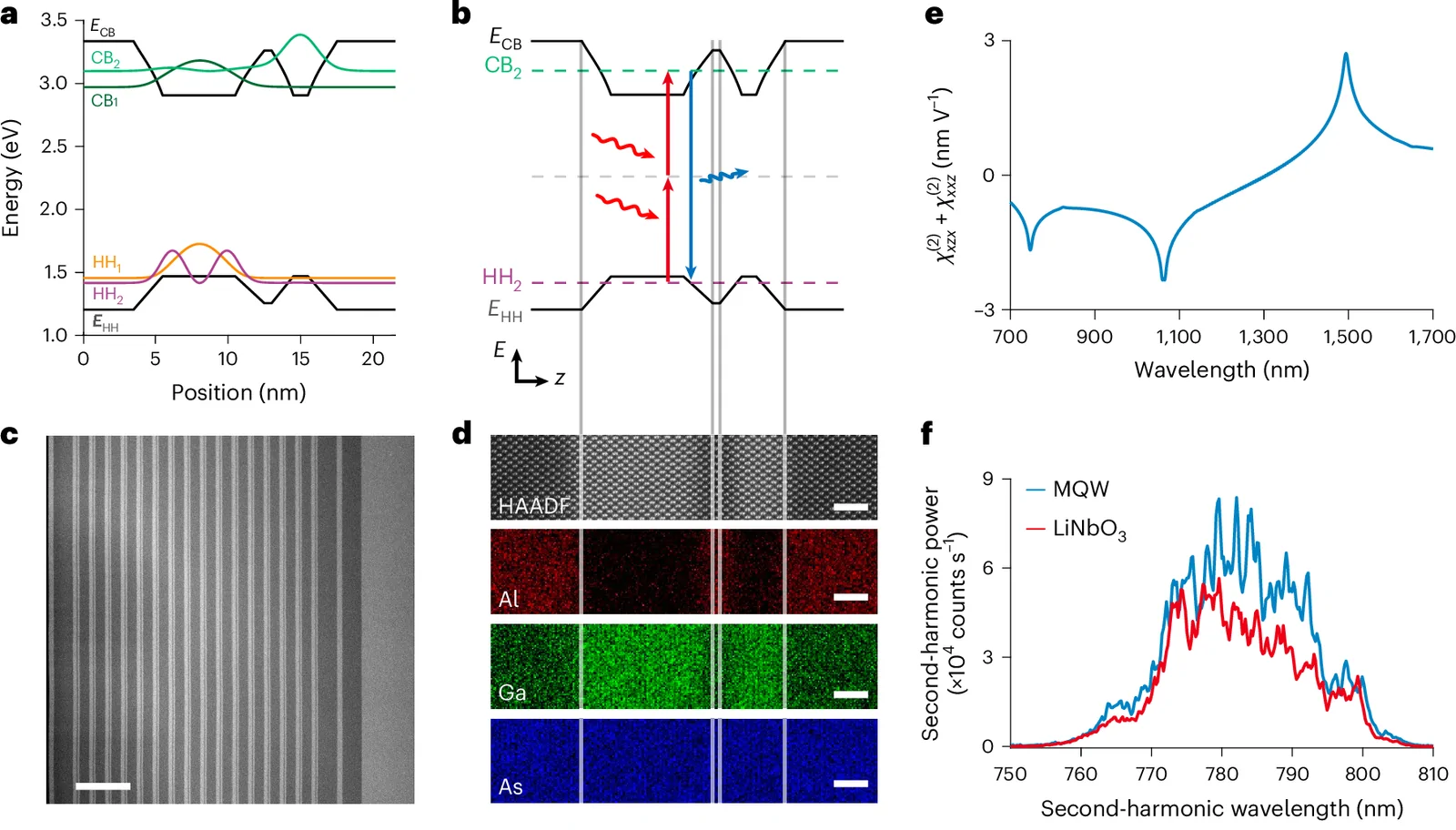
GaAs/AlGaAs heterostructure properties. **a**, Band-edge diagram of a single GaAs/AlGaAs double well showing the energies (*E*_HH_, *E*_CB_) and the probability distributions of the first two heavy-hole (HH_1_, HH_2_) and conduction-band (CB_1_, CB_2_) states. The diagram shows that the heavy-hole states and CB_1_ are confined to the wider quantum well, while CB_2_ is confined to the thinner well. **b**, Band-edge diagram demonstrating the transitions involved in the HH_2_-to-CB_2_ nonlinear enhancement. The asymmetry of the structure leads to an offset between the centroids of the probability distributions of the two states, giving rise to a dipole-like diagonal intersubband matrix element creating a large second-order nonlinearity. **c**, HAADF-STEM image of a heterostructure cross-section showing multiple GaAs/AlGaAs double wells (scale bar, 100 nm). **d**, High-magnification HAADF-STEM image (top) and corresponding EDS maps of Al, Ga and As in a single period of the MQW structure (scale bars, 2 nm). **e**, Simulated second-order susceptibility of the structure in **a** as a function of the pump frequency during second-harmonic generation (smoothed using a third-order Savitzky–Golay filter). **f**, Experimental second-harmonic spectrum generated by the MQW measured at 45° incidence angle and *x*-cut LiNbO_3_ at 0° incidence angle (after subtracting a constant background intensity). In both samples, the nonlinear medium is 0.6 μm thick.

To induce such asymmetry, we couple two GaAs/AlGaAs quantum wells with different widths to form the basis of our heterostructure. Figure 2a shows their band diagram and the probability distributions of the first two heavy-hole and conduction-band states. The second heavy-hole state (HH_2_) is confined in the wider of the two quantum wells, whereas the second conduction-band state (CB_2_) is confined in the thinner well^34^. The HH_2_-to-CB_2_ interband transition is therefore associated with a large electron displacement (towards the right in Fig. 2a) without an opposite-displacement counterpart, substantially enhancing the second-order nonlinear properties of the double well close to the transition energy between the HH_2_ and CB_2_ states.

Interband transitions involving a heavy-hole state require a photon polarized perpendicular to the growth direction (along *x*, *y*) of the layers to satisfy the interband selection rules at the Γ point^36^. One pump photon and the second-harmonic photon share this polarization. To benefit from the tailored asymmetry, which the double well only enforces along the growth direction, the second pump photon must be polarized along the growth direction (*z*)^37^. At the transition energy, the selection rules for the MQW-interband transition yield dominant ${\chi }_{xzx}^{(2)}={\chi }_{xxz}^{(2)}=-{\chi }_{yzy}^{(2)}=-{\chi }_{yyz}^{(2)}$ tensor elements with large magnitudes and opposite signs in the near-infrared region (Supplementary Section 1). To amplify the magnitude of the dominant tensor elements, we adjust the barrier thickness, degree of asymmetry and overall size of the double quantum well. These parameters tune the overlap and centroids of the HH_2_ and CB_2_ probability distributions. Details on the design, growth and characterization are in the Methods.

For our demonstration, we use a 16-period MQW film with a total thickness of 595 nm, bonded to a sapphire substrate with epoxy resin. Neighbouring double wells are separated by a thick AlGaAs barrier to prevent adjacent quantum-well states from hybridizing. Figure 2c,d shows high-angle annular dark-field scanning transmission electron microscopy (HAADF-STEM) and energy-dispersive X-ray spectroscopy (EDS) characterization of the grown heterostructure (Methods). The measured composition profiles reveal non-abrupt GaAs/AlGaAs interfaces. We use this experimentally corrected band profile in the numerical simulations of the nonlinear response, yielding a spectrally resolved ${\chi }_{xzx}^{(2)}(\omega )+{\chi }_{xxz}^{(2)}(\omega )$, shown in Fig. 2e. Simulation details are provided in the Methods and refs. ^34,35^.

We determine $\left\vert {\chi }_{xzx}^{(2)}+{\chi }_{xxz}^{(2)}\right\vert$ by illuminating the heterostructure with *x*-polarized, 70-fs-long laser pulses and comparing its second-harmonic flux at 45° incidence with that from an *x*-cut LiNbO_3_ thin film of equal thickness at normal incidence and otherwise identical experimental conditions. Probing the *x*-based tensor elements is arbitrary, and the *y*-based tensor elements would give an equivalent response. LiNbO_3_ provides a well-characterized, low-loss reference. Figure 2f compares the spectrally resolved second-harmonic fluxes. Calibrating the measurement with the known ${\chi }_{zzz,{\mathrm{LiNbO}}_{3}}^{(2)}$ = 51.9 pm V^−1^ of LiNbO_3_ (Methods), the refractive indices of the samples and the reflection, absorption and thin-film interference in our samples yields a MQW-material-only (intrinsic) second-order susceptibility ${\chi }_{\mathrm{intrinsic},\mathrm{MQW}}^{(2)}=| {\chi }_{xzx}^{(2)}+{\chi }_{xxz}^{(2)}|$ = 1.6 nm V^−1^ at 1.57 μm, four times that of bulk GaAs (${\chi }_{xyz,{\rm{GaAs}}}^{(2)}$ = 370 pm V^−1^; ref. ^38^) and two orders of magnitude larger than that of LiNbO_3_. Neglecting second-harmonic absorption in the analysis of the heterostructure slightly reduces the usable ${\chi }_{{\rm{intrinsic}},{\rm{MQW}}}^{(2)}$ to 1.2 nm V^−1^ at 1.57 μm. Notably, despite its large ${\chi }_{{\rm{intrinsic}},{\rm{MQW}}}^{(2)}$, the bare MQW film produces second-harmonic powers comparable only to the similarly thick LiNbO_3_ film, highlighting the need for a different excitation scheme in the case of MQW-interband-driven nonlinearities.

The measured $\left\vert {\chi }_{{\rm{intrinsic}},{\rm{MQW}}}^{(2)}\right\vert$ reaches ~60% of the numerical prediction shown in Fig. 2e. This difference is explained by the relatively thick AlGaAs barriers separating subsequent double wells, which do not contribute to enhancing the nonlinearity, lowering the average response of the thin film. Even higher experimental ${\chi }_{{\rm{intrinsic}},{\rm{MQW}}}^{(2)}$ values can be achieved by reducing this barrier thickness, or by improving the MQW interface sharpness through the introduction of growth interruptions during molecular beam epitaxy^35^.

## Metasurface design

We choose to access the ${\chi }_{xzx}^{(2)}$ and ${\chi }_{xxz}^{(2)}$ tensor elements of the MQW film, and employ metasurface-enabled resonances to create the necessary light polarization components and enhance the electromagnetic field in the heterostructure. Metasurfaces can sustain a variety of resonant responses, from localized Mie modes of individual nanostructures to collective, non-local effects arising from periodic arrays of scatterers. Here we use GMRs, a non-local resonance type^39–42^.

Conceptually, GMRs can be understood in terms of a grating coupler to a waveguide: a subwavelength grating diffracts incident light into a high-index slab. If the diffracted propagation angle of the light allows total internal reflection in the layer and the light accumulates the correct round-trip phase, the light couples to a guided mode and is later out-coupled by the same grating. Large gratings with weak radiative coupling yield sharp resonances. Because the resonant modes propagate along the waveguide, they can create strong *E*_*x*_ and *E*_*z*_ fields, which can in turn drive the ${\chi }_{xzx}^{(2)},{\chi }_{xxz}^{(2)}$ nonlinearity.

To realize a GMR at a pump wavelength of 1.58 μm, we use a TiO_2_ nanopillar array, on top of the heterostructure, to act as the waveguide. We tune the periodicities, pillar dimensions and height to provide the correct light polarization components *E*_*x*,*ω*_, *E*_*z*,*ω*_ and a high quality factor, thereby providing a long resonance lifetime and strong field enhancement. To ensure efficient second-harmonic generation, we then search for a high modal overlap factor, *β*, between the pump fields and the complex-conjugated second-harmonic field E*_x,2*ω*_ in the heterostructure^13,43–45^:

1 $$\beta \propto | {\int }_{\mathrm{MQW}}{E}_{x,\omega }{E}_{z,\omega }{E}_{x,2\omega }^{* }{\mathrm{d}}^{3}x|$$

**Fig. 3:**
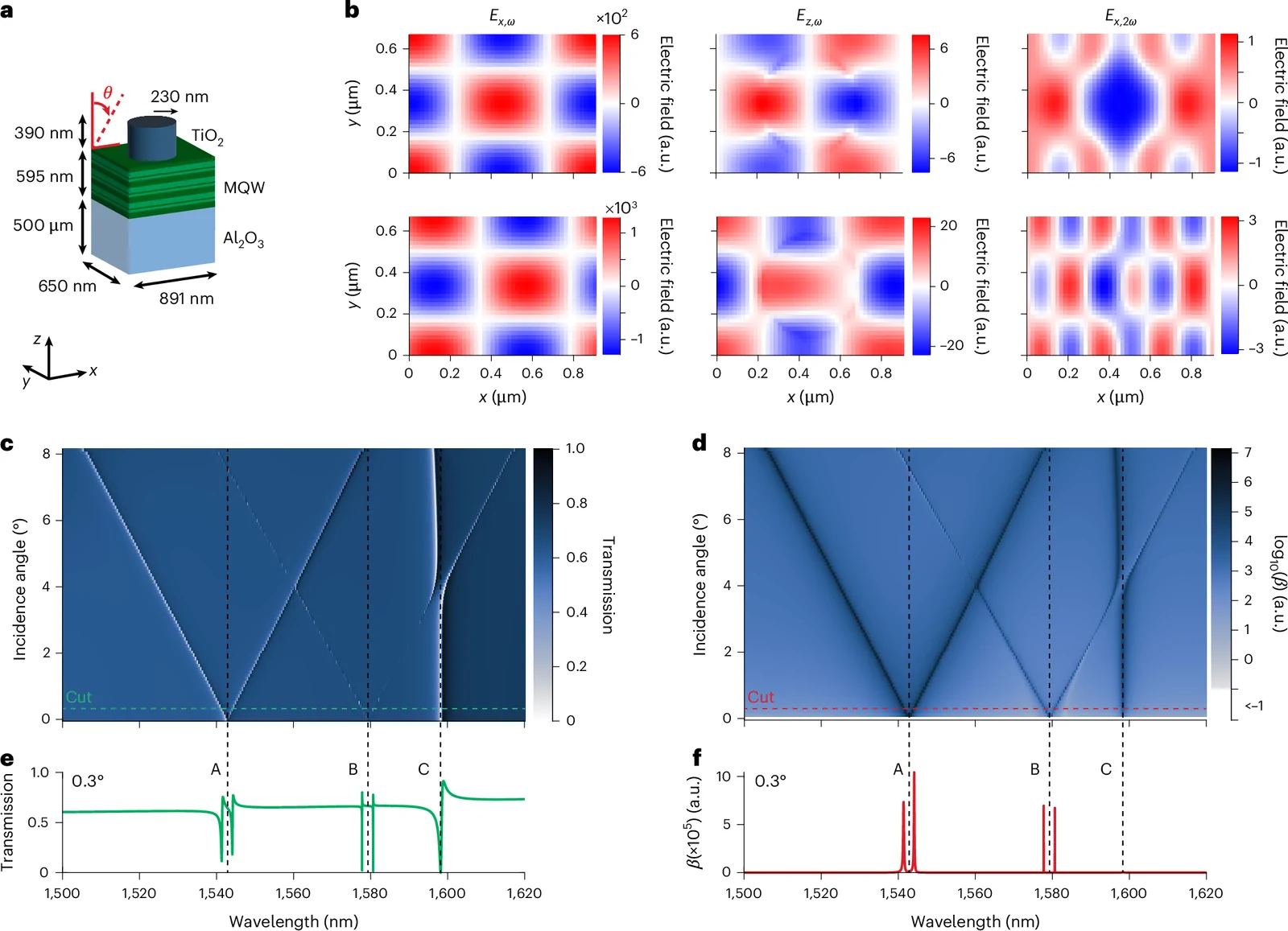
Metasurface design and device simulations. **a**, Metasurface unit cell with dimensions. The sample is rotated about the *y* axis, introducing a non-zero *k*_*x*_ momentum component. **b**, Field distribution in the waveguide at resonance. The panels show the spatial (*x*–*y*) profile of the simulated fields in the waveguide, summed along the growth direction (*z*; the spatial (*y*–*z*) distribution is in Supplementary Fig. 1). We show the *x* and *z* components of the pump field (left columns) and the *x* component of the second-harmonic field (right column) for normal, 0° incidence angle (top row) and 0.3° incidence angle (bottom row) for the left branch of resonance B (described in **c** and **d**). For normal incidence, the symmetry of the fields yields a vanishing modal overlap, *β*. **c**, Simulated transmission spectrum of the sample for *x*-polarized light incident at angles between 0° and 8°, introduced by tilting the sample about the *y* axis. We observe three GMRs, labelled A, B and C. Resonances A and B split spectrally for finite incidence angles. Resonance C requires a finite incidence angle from tilting about the *x* axis to spectrally split. **d**, Simulated modal overlap spectrum (*β* as a function of *λ*_0_, where *λ*_0_ is the vacuum wavelength of the pump) of the sample for *x*-polarized light incident at angles between 0° and 8°, demonstrating negligible overlap at normal incidence. At finite incidence angles, the breaking of the electric field symmetry (**b**) enables large peaks in the overlap that closely follow the spectral positions of the GMRs. **e**, Simulated transmission spectrum for *x*-polarized light incident at 0.3°, corresponding to the cut marked in **c**. **f**, Simulated modal overlap spectrum for *x*-polarized light incident at 0.3°, corresponding to the cut marked in **d**. The electric field of resonance C is largely confined to in-plane components, thereby suppressing the modal overlap compared with resonances A and B.

Figure 3a illustrates the resulting device unit cell. Figure 3c,e shows its spectrally resolved transmission for incidence angles between 0° and 8° as the sample is rotated about the *y* axis, and Fig. 3d,f shows the corresponding modal overlap. The displayed spectral range contains three GMRs, labelled A, B and C.

An inversion-symmetric grating provides equal and opposite in-plane momentum, $\pm m{\mathbf{k}}_{{\rm{g}}}$, where *m* is the grating order. At normal incidence, the two counter-propagating GMRs are therefore degenerate, producing a single sharp transmission dip with waveguide momentum ${\mathbf{k}}_{xy,\mathrm{waveguide}}=\pm m{\mathbf{k}}_{{\rm{g}}}$. Tilting the sample adds incident in-plane momentum, ${\mathbf{k}}_{xy,{\rm{inc}}}$, so the resonance condition becomes ${\mathbf{k}}_{xy,\mathrm{waveguide}}={\mathbf{k}}_{xy,\mathrm{inc}}\pm m{\mathbf{k}}_{{\rm{g}}}$. The two branches then satisfy phase matching at different frequencies, causing the angle-dependent splitting observed for resonances A and B in Fig. 3e. Resonance C couples to modes propagating along the orthogonal lattice direction and would split under rotation about the *x* axis.

In spite of the presence of the required pump fields *E*_*x*,*ω*_ and *E*_*z*,*ω*_, at normal incidence, these fields possess opposite parity (illustrated by the field cross-sections in Fig. 3b, top row), resulting in a vanishing nonlinear overlap integral. As a result, the corresponding nonlinear process is not simply weak, but symmetry forbidden (Supplementary Section 2). Accessing these tensor elements therefore requires not only field enhancement, but also intentional symmetry breaking of the optical mode. The introduction of a small incidence angle breaks the symmetry of the structure (Fig. 3b, bottom row) and creates a large overlap between the required field components (Fig. 3d). The wavelength of maximal overlap closely follows the GMR position (compare Fig. 3c,d), enabling device tuning. Figure 3e,f shows the spectral cut of transmission and modal overlap at 0.3°, where most experimental characterization was performed.

## Metasurface-enhanced nonlinear response

Following the schematic in Fig. 3a, we fabricate a 600 μm × 600 μm TiO_2_ metasurface on the surface of the MQW structure. Fabrication details are given in the Methods.

**Fig. 4:**
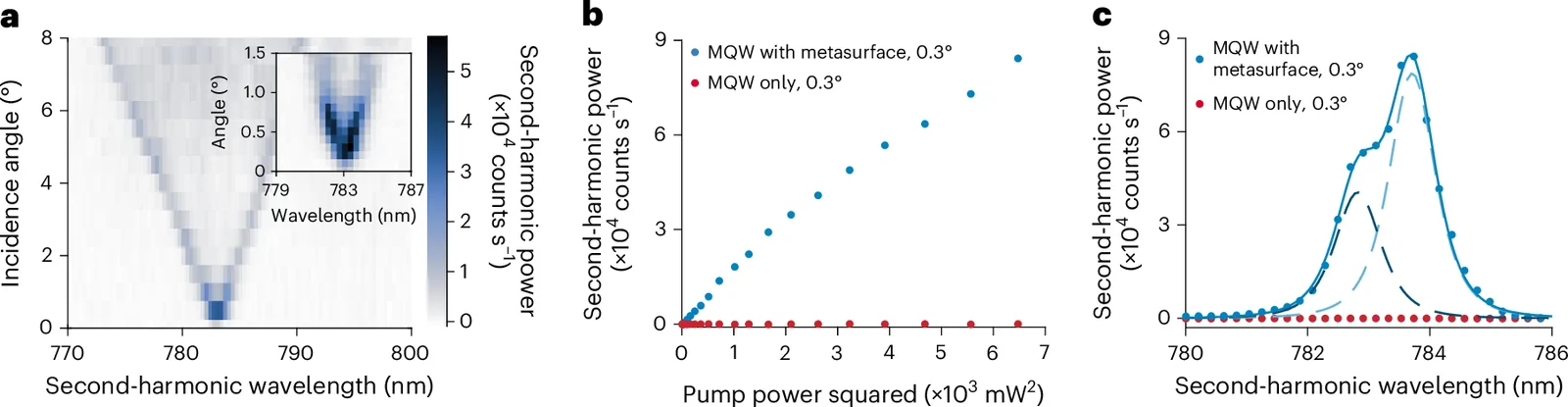
Measured nonlinear device response. **a**, Experimental pump-incidence-angle-resolved second-harmonic spectra measured at a pump power of 41 mW. The second-harmonic maxima follow the angular evolution of the GMR branches (compare with Fig. 3c). The inset image shows a high-resolution scan from 0° to 1.5°. At normal incidence, the measurement shows negligible second-harmonic response, in accordance with the simulated *β*. **b**, Measured second-harmonic power as a function of the squared average pump power, demonstrating a quadratic dependence at lower pump powers. **c**, Second-harmonic spectrum measured at a pump power of 80 mW for the bare MQW and the MQW with a surface-patterned metasurface, both at 0.3° angle of incidence. The second-harmonic spectrum generated by the metasurface-MQW device is fitted with the sum of two squared Lorentzians. Dashed lines show the individual squared Lorentzian fits, and the solid line shows the sum. Detector noise has been removed by subtracting a constant background intensity from all measurements.

We illuminate the sample with *x*-polarized light and rotate it around the *y* axis (Supplementary Fig. 2 has a schematic of the experimental set-up) and measure the second-harmonic response in transmission, shown in Fig. 4. Resonance B, for which the simulations predict the highest quality factor, generates the highest second-harmonic power; thus, we concentrate the following discussion on it. Our measurements show the realized resonance at 1.567 μm pump wavelength (that is, it is shifted by 13 nm compared with the simulations, explainable by a less than 5% deviation in the heterostructure’s thickness or refractive index). Linear transmission measurements of the metasurface and nonlinear co-polarization and cross-polarization measurements are provided in Supplementary Figs. 3 and 4.

Figure 4a shows the experimentally measured second-harmonic power created by the metasurface-heterostructure device for incidence angles varying from 0° to 8°. Absolute second-harmonic powers and normalized conversion efficiencies are calibrated as described in Supplementary Section 3. At normal incidence, we measure a negligible second-harmonic intensity, consistent with the vanishing overlap observed in the simulations. At finite incidence angles, second-harmonic radiation appears at distinct wavelengths. As predicted by the calculated *β*, the second-harmonic peaks’ spectral positions follow the tunable angle splitting of the GMRs at the pump wavelength.

The largest second-harmonic response is observed at small incidence angles. This is a result of two contributing factors: (1) the resonance quality factor decreases with increasing angle, reducing field enhancement, and (2) at small angles, the two angle-split GMR branches remain spectrally overlapped, contributing additively to the second-harmonic signal. At larger angles, the branches separate and contribute independently, reducing the combined response. We measure the peak second-harmonic power at 0.3° incidence angle, which scales quadratically with the pump power at low intensities (Fig. 4b), confirming the second-order nature of the process.

At high powers, the second-harmonic response shows a slight deviation from quadratic scaling. We attribute this to a broadening of the resonance (Supplementary Fig. 5). We presume this stems from the spatially dependent pump beam’s thermo-optic effect, noting that only 1.3% of the pump beam’s spectrum is used by the GMR, yet the full spectrum contributes thermo-optically. By comparison, the unpatterned heterostructure shows no measurable response under the same experimental conditions (Fig. 4b,c). This verifies that the metasurface enables the ${\chi }_{xzx}^{(2)}$-driven and ${\chi }_{xxz}^{(2)}$-driven second-harmonic generation.

Even for the patterned sample, as the incidence angle increases, the oblique incidence introduces a larger *E*_*z*_ to the heterostructure, which is evident from a broad, low-intensity, sum-frequency background emerging gradually in Fig. 4a. At 0.3° incidence angle, the absence of a broad background confirms that the field components required for second-harmonic generation are introduced by the narrow metasurface resonance. Furthermore, each GMR branch of resonance B contributes independently to second-harmonic generation, as evidenced by the lack of a central peak corresponding to sum-frequency generation from the combination of photons in the different resonance branches. The total second-harmonic response is therefore well described as the sum of two independent resonances, allowing us to fit the second-harmonic spectrum with two squared Lorentzians (Fig. 4c). This yields a 0.89 nm second-harmonic line-width and a 1.39 nm pump resonance line-width at 0.3° incidence angle. This corresponds to a quality factor of 1,125 and confirms the high quality of the resonance.

To evaluate the effective nonlinear tensor element of our metasurface-coupled heterostructure, we compare its experimentally measured second-harmonic flux (at 0.3° incidence angle) with that generated by the unpatterned heterostructure (at 45° incidence angle). We reproduce the data by modelling second-harmonic and sum-frequency generation for both measurements, taking into account the heterostructure’s refractive index, thin-film interference and reflections, and the metasurface’s spectral acceptance versus the pump laser’s bandwidth (which is ~100 times larger than the GMR bandwidth; Methods).

At 1.57 μm, we find that the metasurface enhances the pump’s *E*_*x*_*E*_*z*_ field product in the heterostructure by ~57 times compared with the *E*_*x*_*E*_*z*_ field product present in the heterostructure at 45° incidence. Furthermore, our model shows an ~11.5 times enhancement when comparing the metasurface-generated *E*_*x*_*E*_*z*_ field product in the heterostructure with the *E*_*x*_*E*_*x*_ field product present in the heterostructure at normal incidence (which does not require polarization conversion).

As the metasurface allows for the sample to be used with *x*-polarized light at normal incidence, this yields an effective, usable ${\chi }_{\mathrm{eff},\mathrm{MQW}+\mathrm{MS}}^{(2)}={\chi }_{xxx,\mathrm{MQW}+\mathrm{MS}}^{(2)}$ ≈ 14 nm V^−1^, more than 270 times larger than the largest *χ*^(2)^ tensor element of LiNbO_3_. Note that correcting for absorption of the second-harmonic radiation in the heterostructure, one obtains a nonlinearity of 17 nm V^−1^. Here we report the lower nonlinear susceptibility, as it represents the useful output from the device.

Optimal use of any resonance-enhanced device requires matching the resonance bandwidth to the pump source, as all spectral content outside of the resonance bandwidth is discarded. Here, the MQW design allows us to spectrally tune the material resonance, and the spectral position and line-width of the GMR can be altered by optimizing the metasurface design. This enables closed-loop design and optimization of nonlinear devices where every aspect of the polarization is tailored to the intended application.

## Conclusions

By coupling interband-engineered MQWs to high-quality-factor metasurface resonances, we demonstrate a co-design approach that simultaneously enhances the intrinsic nonlinear susceptibility and provides optical access to non-trivial tensor elements at near-infrared wavelengths. While the GaAs/AlGaAs heterostructure exhibits a large ${\chi }_{{\rm{intrinsic}},{\rm{MQW}}}^{(2)}$, this response remains inefficient under conventional free-space excitation, resulting in second-harmonic generation comparable to that of established materials such as LiNbO_3_. By introducing symmetry-broken, resonantly enhanced electromagnetic fields, the metasurface enables efficient coupling to these intrinsic nonlinearities and provides an additional enhancement of the nonlinear response. This results in a transition from a material with large but underutilized nonlinear properties to a functional nanophotonic platform with strongly enhanced effective nonlinearity. Even larger nonlinear responses can be achieved by further optimizing fabrication and expanding the design space of both the heterostructure and the metasurface.

The versatility of the metasurface can extend our demonstration to a range of applications. While we use a non-local metasurface for field enhancement and polarization conversion, other metasurfaces can spatially engineer the nonlinear output by controlling the propagation phase or geometric phase. Beyond second-harmonic generation, this concept can also be used for difference-frequency generation, and can enable entangled photon generation through spontaneous parametric downconversion. As the device is entirely based on dielectric and semiconducting materials, it combines a high damage threshold and foundry compatibility. The considerable *χ*^(2)^ magnitude increase with respect to the bulk third-order nonlinear susceptibility, *χ*^(3)^, enables devices with less three-photon absorption, hence allowing for further miniaturization or operation under relaxed phase-matching conditions compared with their bulk-crystal counterparts.

The combination of plasmonic metasurfaces and quantum wells has proven powerful in the mid-infrared^12^, and the present demonstration establishes the near-infrared-to-visible counterpart. Furthermore, the presented approach to band engineering can be extended to higher-order nonlinearities, enabling Kerr-based nonlinear devices with tailored nonlinear susceptibilities. The work highlights the power of uniting band-structure engineering with resonant nanophotonics and opens doors for high-efficiency devices at wavelengths of great technological importance.

## Methods

### Quantum-mechanical simulation and *χ*^(2)^ calculation

Starting from the general form of the dipole matrix formalism for the second-order nonlinear susceptibility^32,38,46^, the expression for the electron (e) and the heavy-hole (hh) susceptibility of the resonant tensor element can be expressed as

2 $$\begin{array}{l}{\chi }_{xzx,{\rm{e}}}^{(2)}({\omega }_{1}+{\omega }_{2},{\omega }_{1},{\omega }_{2})=\displaystyle\frac{{N}_{z}{e}^{3}{r}_{{\rm{e}},\mathrm{hh}}^{2}}{6{\epsilon }_{0}{\hslash }^{2}}\sum _{{k}_{\parallel }}\sum _{m,n}\sum _{l}\\\qquad\qquad\qquad\qquad\qquad\left(\displaystyle\frac{\langle {\psi }_{\mathrm{hh},m}| {\psi }_{{\rm{e}},n}\rangle \langle {\psi }_{{\rm{e}},n}| z| {\psi }_{{\rm{e}},l}\rangle \langle {\psi }_{{\rm{e}},l}| {\psi }_{\mathrm{hh},m}\rangle }{({\omega }_{\mathrm{hh},m}^{{\rm{e}},n}({k}_{\parallel })-{\omega }_{1}-{\omega }_{2}+i\Gamma )({\omega }_{\mathrm{hh},m}^{{\rm{e}},l}({k}_{\parallel })-{\omega }_{1}+i\Gamma )}\right)\end{array}$$

3 $$\begin{array}{l}{\chi }_{xzx,\mathrm{hh}}^{(2)}({\omega }_{1}+{\omega }_{2},{\omega }_{1},{\omega }_{2})=\displaystyle\frac{{N}_{z}{e}^{3}{r}_{{\rm{e}},\mathrm{hh}}^{2}}{6{\epsilon }_{0}{\hslash }^{2}}\sum _{{k}_{\parallel }}\sum _{m,n}\sum _{l}\\\qquad\qquad\qquad\qquad\qquad\left(-\displaystyle\frac{\langle {\psi }_{{\rm{e}},n}| {\psi }_{\mathrm{hh},m}\rangle \langle {\psi }_{\mathrm{hh},m}| z| {\psi }_{\mathrm{hh},l}\rangle \langle {\psi }_{\mathrm{hh},l}| {\psi }_{{\rm{e}},n}\rangle }{({\omega }_{\mathrm{hh},m}^{{\rm{e}},n}({k}_{\parallel })-{\omega }_{1}-{\omega }_{2}+i\Gamma )({\omega }_{\mathrm{hh},l}^{{\rm{e}},n}({k}_{\parallel })-{\omega }_{1}+i\Gamma )}\right)\end{array}$$

where [*m*, *n*, *l*] denote bound states in the conduction and heavy-hole bands, *ψ*_e,hh_ are the corresponding envelope wavefunctions, *ω*_1,2_ denote the input photon frequencies, ${\omega }_{\mathrm{hh};m,l}^{{\rm{e}};n,l}$ represent interband transition energies, *Γ* is the phenomenological broadening parameter (here set to 5 meV), *k*_∣∣_ denotes the in-plane momentum, *N*_*z*_ is the spin degeneracy, *e* is the elementary charge, *ℏ* is the reduced Planck constant and *i* is the imaginary unit. The quantity ${r}_{{\rm{e}},\mathrm{hh}}=\langle {u}_{{\rm{e}}}| r| {u}_{\mathrm{hh}}\rangle$ is the interband dipole matrix element where *r* is the position operator, and *u*_e_ and *u*_hh_ are the cell-periodic Bloch functions of the electron and heavy hole states. The electron and heavy-hole susceptibilities contain the intersubband matrix elements of the electrons and heavy holes, respectively.

The envelope functions of the coupled quantum wells were obtained from self-consistent Schrödinger–Poisson simulations performed with Nextnano^47^. The interband matrix element *r*_e,hh_ for GaAs was calculated using density functional theory in the Vienna Ab initio Simulation Package with the HSE06 hybrid functional. The summation over in-plane *k* states was evaluated by converting it to a two-dimensional integral over (*k*_*x*_, *k*_*y*_), truncated at one-tenth of the Brillouin zone, beyond which the contribution to *χ*^(2)^ was found to be negligible^34^.

### Material growth and substrate transfer

The III–V heterostructures were grown on semi-insulating GaAs(100) wafers using molecular beam epitaxy in a Varian Gen II system. The system was equipped with solid-source thermal effusion cells for Al and Ga and a solid-source valved cracker for As. The growth temperature was maintained at 600 °C, monitored by band-edge thermometry. AlGaAs was grown at 1.85 Å s^−1^ and GaAs was grown at 0.83 Å s^−1^ under a ×15 As overpressure. The heterostructure contains 16 periods, each comprising two AlGaAs barrier layers (shown in bold) and two GaAs quantum wells, following the layer sequence (in nanometres) of **18.2**/7.1/**1.8**/2.9. Etch-stop layers were grown to enable subsequent flip-chip transfer to Al_2_O_3_ substrates. The substrate transfer was done by bonding the heterostructure to Al_2_O_3_ with 353ND, EPO-TEK epoxy resin, followed by mechanical lapping for etching of the GaAs substrate and wet etch removal of the etch-stop layers (Supplementary Fig. 6).

### Electron microscopy characterization of MQW film

Using a focused ion beam, lamellae for STEM were prepared and subsequently fine-polished with Ar using a nano-mill. Using a double-aberration-corrected JEOL ARM-300CF microscope operating at 300 kV, STEM images were acquired. We performed the *z*-contrast HAADF-STEM imaging with a probe convergence semi-angle of 25.7 mrad and an inner collection angle of 53 rad.

EDS maps were taken with dual 100 mm^2^ Si drift detectors. The EDS maps were constructed by summing 100 drift-corrected scans, each with a dwell time and step size of 10 ms and 52 pm, respectively. Subsequently, the composition profiles of Ga and Al were fitted as piecewise linear functions in the growth direction to be used for the quantum-mechanical simulations of the bound-state wavefunctions and energy levels of the grown material.

### Metasurface simulations

The electromagnetic simulations were carried out with rigorous coupled-wave analysis using GRCWA^48^. The structural parameters used in the simulations were a pillar height of 390 nm, a radius of 230 nm, an *x* periodicity of 891 nm and a *y* periodicity of 650 nm, with p-polarized incident light. The refractive index of the TiO_2_ was measured by ellipsometry of an amorphous TiO_2_ thin film deposited by atomic layer deposition under the same conditions as the pillars of the metasurface. The refractive index of the MQW stack was determined by ellipsometry of the equivalent GaAs/AlGaAs stack on a GaAs substrate. Measured refractive indices are in Supplementary Figs. 7 and 8. In the simulations, the MQW stack was treated as a single material layer with the measured refractive index. Equivalent simulations were completed for the pump and second-harmonic wavelengths, and the corresponding pairs were used to compute the modal overlap.

### Metasurface fabrication

Undiluted positive-tone electron-beam lithography resist, ZEP 520A, was spin-coated at 3,800 rpm for 45 s. The sample was pre-baked on a hotplate at 90 °C for 3 min, followed by a hotplate at 180 °C for 3 min. A conductive polymer (Showka Denko ESPACER 300) was spun at 1,500 rpm for 45 s to avoid charging effects. The resist was patterned using electron-beam lithography (Elionix BODEN 150) with an acceleration voltage of 150 kV and a current of 1 nA. The sample was developed with *o*-xylene under gentle agitation. TiO_2_ was deposited by atomic layer deposition (Savannah, by Cambridge NanoTech), followed by reactive-ion etching (Oxford PlasmaPro 100 Cobra 300) of overgrown TiO_2_. Finally, the resist was removed with Remover PG. A schematic is in Supplementary Fig. 9.

### Experimental characterization

Linear optical characterization of the unpatterned GaAs/AlGaAs heterostructure was done using a Cary 7000 Universal Measurement Spectrophotometer. Linear and nonlinear characterization of the metasurface device was done using a MenloSystems ELMO femtosecond erbium laser centred at 1,560 nm with an average output power of 330 mW, pulse duration of 70 fs and repetition rate of 100 MHz. The linear signal was collected with an Anritsu MA9710B optical spectrum analyser, and the nonlinear response was measured with an Andor SR-500i-B2-R spectrometer with an Andor Newton 971 EMCCD detector. The optical characterization set-up is shown in detail in Supplementary Fig. 2.

### Measurement of ${{\boldsymbol{\chi }}}_{{\boldsymbol{x}}{\boldsymbol{z}}{\boldsymbol{x}}}^{({\bf{2}})}({\boldsymbol{\omega }})+{{\boldsymbol{\chi }}}_{{\bf{x}}{\bf{x}}{\bf{z}}}^{({\bf{2}})}({\boldsymbol{\omega }})$ by comparison with LiNbO_3_

To determine the magnitude of ${\chi }_{xzx}^{(2)}(\omega )+{\chi }_{xxz}^{(2)}(\omega )$ in the MQW, we performed relative measurements against a reference sample. A 0.60-μm-thick *x*-cut congruent thin film of LiNbO_3_ on sapphire was measured at normal incidence, and a 0.60-μm-thick MQW film on sapphire substrate was measured at 45° incidence. The samples were excited from the substrate side under equal pumping conditions, and the transmitted pump and generated second-harmonic spectra were recorded. Second-order nonlinear susceptibility ${\chi }_{zzz}^{(2)}(1,567\,{\mathrm{nm}})$ values measured close to a 1,567 nm pump wavelength for LiNbO_3_ are lacking; the measured value referred to most often in the literature is ${\chi }_{zzz}^{(2)}(1,064\,\mathrm{nm})$= 54.4 pm V^−1^ (refs. ^49–51^).

Applying Miller’s rule^52^ to this value predicts ${\chi }_{zzz}^{(2)}(1,567\,\mathrm{nm})$= 51.9 pm V^−1^. Careful measurements by Shoji et al. found ${\chi }_{zzz}^{(2)}(852\,\mathrm{nm})$ = 51.4 pm V^−1^, ${\chi }_{zzz}^{(2)}(1,064\,\mathrm{nm})$ = 50.4 pm V^−1^ and ${\chi }_{{zzz}}^{(2)}(1,313\,\mathrm{nm})$ = 39.0 pm V^−1^ (ref. ^53^); however, the latter value deviates considerably from the Miller’s rule expectation. The highest value, ${\chi }_{zzz}^{(2)}(1,064\,\mathrm{nm})$ = 83.4 pm V^−1^, was reported for stoichiometric LiNbO_3_, which contains a higher lithium fraction than congruent LiNbO_3_ (refs. ^50,54^). For consistency with the current body of literature, this work uses ${\chi }_{zzz}^{(2)}(1,567\,\mathrm{nm})$= 51.9 pm V^−1^.

To account for reflections at the sample interfaces and thin-film interference, we model propagation into our experimental structures, including the respective incidence angle, using finite-difference time-domain simulations (Flexcompute Tidy3D; https://github.com/marcus-o/linbo_mqw_comparison). To model the experimental pump-pulse spectrum, we Fourier transform the incident laser pulses’ spectrum, inject the resulting electric field using a time-dependent source and record the polarization-resolved time-dependent electric field *E*_*x*_(*t*) and *E*_*z*_(*t*) at the centre position of our thin films.

The use of a time-dependent electric field *E*(*t*) inherently accounts for the pulsed nature of the excitation and avoids the need for a continuous-wave approximation. In particular, the time-dependent second-order nonlinear polarization *P*^(2)^ is computed as *P*^(2)^(*t*) ∝ *χ*^(2)^*E*^2^(*t*), such that the temporal profile of the pulse, including its peak intensity, is explicitly captured in the simulation. As the extraction of *χ*^(2)^ is performed through a relative calibration against a LiNbO_3_ reference measured under identical excitation conditions, the absolute amplitude of the electric field cancels. Consequently, the extracted *χ*^(2)^ does not depend on whether the pump is expressed in terms of peak power or average power.

Whereas the frequency dependence of the nonlinear response of LiNbO_3_ is small in our laser’s wavelength range, we account for the heterostructure’s frequency dependence by modelling *χ*^(2)^ in the time domain as the product of two independent and exponentially decaying (lifetime or dephasing time, 30 fs) oscillators. The coherence lengths for second-harmonic generation from 1,550 nm to 775 nm wavelength are 1.8 μm in the heterostructure and 9.6 μm in LiNbO_3_. As the samples are considerably thinner, phase matching and pump depletion are negligible, and we assume that sum-frequency radiation builds up coherently along the sample. We correct for the sum-frequency propagation and absorption (*n*_780nm_ = 3.39 + 0.11*i* from ellipsometry measurements) outside of the samples using a finite-difference time-domain simulation. Under the assumption that the laser pulses’ temporal/spectral phases do not possess strong second-order (chirp) or higher-order phase components in the samples, the simulated and experimentally measured sum-frequency spectra match^55^ (Supplementary Fig. 7).

Finally, we scale the heterostructure’s ${\chi }_{xzx}^{(2)}(\omega )+{\chi }_{xxz}^{(2)}(\omega )$ so the simulated and experimentally measured sum-frequency fluxes match for LiNbO_3_ and the heterostructure (Supplementary Fig. 10). This relative approach removes the need for an absolute pump intensity calibration and enables a direct comparison between the MQW and LiNbO_3_ under consistent experimental and numerical conditions.

### Measurement of resonant second-harmonic generation enhancement

To measure the resonant enhancement by the GMR, we compare the second-harmonic flux generated by the metasurface sample at a 0.3° incidence angle with the sum-frequency generated by the bare MQW film at 45° incidence angle using otherwise identical excitation conditions.

Experimentally, for the metasurface sample at 0.3° incidence angle, we observe no sum-frequency radiation generated by mixing pump photons from different branches of the GMR (which would be observable as additional peaks in the sum-frequency spectrum; Fig. 4a) and no sum-frequency radiation generated by mixing one GMR photon with a non-resonant photon (which would be observable as a broad sum-frequency background; Fig. 4a). Therefore, we fit the generated second-harmonic radiation using the sum of two squared Lorentzian profiles (Fig. 4c) to extract the resonance frequencies and widths of the GMR branches.

We account for the effects of the resonant metasurface in the simulation procedure detailed above by applying a Lorentzian filter to the electric field in the nonlinear layer before calculating sum-frequency generation. The filter uses the experimentally determined centre frequency and width. We then scale the *E*_*x*_(*t*)*E*_*z*_(*t*) product in the heterostructure until the simulated and experimentally measured sum-frequency spectra match those of the metasurface-covered and bare heterostructures, removing the need for an absolute pump intensity calibration.

## Online content

Any methods, additional references, Nature Portfolio reporting summaries, source data, extended data, supplementary information, acknowledgements, peer review information; details of author contributions and competing interests; and statements of data and code availability are available at https://doi.org/10.1038/s41565-026-02268-0.

## Supplementary information

Supplementary Information Supplementary Sections 1–3 and Figs. 1–10.

Peer Review File

## Data Availability

The data supporting the findings of this study are available via Zenodo at https://doi.org/10.5281/zenodo.21246501 (ref. ^56^).

## Code availability

Example data analysis code used in this study is available at https://github.com/marcus-o/linbo_mqw_comparison.
