# Supplementary material for: Quantum-well metasurface for free-space-accessible enhanced nonlinear polarization
Source: Nat Nanotechnol. 2026 Sep 2;21(11):1558–64. doi: 10.1038/s41565-026-02268-0 (PMC13754578; doi:10.1038/s41565-026-02268-0)
Supplement: Supplementary file 1 [file 41565_2026_2268_MOESM1_ESM.pdf]

nature nanotechnology

Supplementary information https://doi.org/10.1038/s41565-026-02268-0

Quantum-well metasurface for free-space-accessible enhanced nonlinear polarization

In the format provided by the authors and unedited

Contents

1 Aspects of Multi-Quantum-Well-Interband-Driven Second Harmonic Generation 1

2 Symmetry Constraints on Nonlinear Modal Overlap 2

2.1 Mode Symmetry at Normal Incidence 2

2.2 Symmetry Breaking 2

3 Second Harmonic Generation Conversion Efficiency 3

4 Supplementary Figures 4

1 Aspects of Multi-Quantum-Well-Interband-Driven Second Harmonic Generation

We achieve the enhanced second order nonlinear susceptibility through interband-transition engineering using multi-quantum-well (MQW) states. The heterostructure is grown in the z direction, and consists of quasi-infinite GaAs/AlGaAs layers along the in-plane x and y directions. Due to its x-y translational symmetry, the heterostructure can only control the spatial position of states, and thus electron motion, along the growth direction. The MQW inherits the in-plane symmetry of the zincblende GaAs/AlGaAs crystal structure, which belongs to point group $\bar{4}3m$. While the heterostructure breaks symmetry along the growth direction z ([001]), the in-plane directions x ([100]) and y ([010]) remain physically equivalent at the material level.

Generally, the second order susceptibility, $\chi^{(2)}_{ijk}(\omega_3, \omega_1, \omega_2)$, depends on the frequencies of three involved photons; however, choosing two photon frequencies determines the third photon's frequency via energy conservation, i.e., $\omega_3 = \omega_1 + \omega_2$. In transparent bulk crystals, when all frequencies are far from resonance (i.e., when absorption and dispersion are negligible), symmetries reduce the number of independent $\chi^{(2)}_{ijk}(\omega_1 + \omega_2, \omega_1, \omega_2)$ tensor elements. This simplification does not directly apply here because the MQW response is resonantly enhanced. However, the second order nonlinear susceptibility responsible for second harmonic generation only depends on the frequency of the incident photons $\chi^{(2)}_{ijk}(\omega) = \chi^{(2)}_{ijk}(2\omega, \omega, \omega)$. In this case, the choice of $\chi_{ijk}(\omega)$ is ambiguous because the $\chi^{(2)}_{\text{intrinsic, MQW}}(\omega)$ relevant for second harmonic generation remains unchanged as long as $\chi^{(2)}_{ijk}(\omega) + \chi^{(2)}_{ikj}(\omega)$ remains constant. We enforce intrinsic permutation symmetry for the two pump field indices j, k, yielding $\chi^{(2)}_{ijk}(\omega) = \chi^{(2)}_{ikj}(\omega)$. Note that the equality is untrue for sum-frequency generation, where $\omega_1 \neq \omega_2$.

2 Symmetry Constraints on Nonlinear Modal Overlap

The efficiency of second harmonic generation in the metasurface-coupled MQW system is governed by the nonlinear modal overlap between the fundamental and second harmonic fields. In the electric dipole approximation, the nonlinear polarization is given by

$$P_i(2\omega) = \epsilon_0 \sum_{j,k} \chi^{(2)}_{ijk} E_j(\omega) E_k(\omega) \tag{1}$$

and the generated second harmonic field scales with the overlap between this nonlinear polarization and the radiating mode,

$$\beta \propto \left| \int_{\text{MQW}} E_{x,\omega} E_{z,\omega} E^*_{x,2\omega} d^3x \right| \tag{2}$$

2.1 Mode Symmetry at Normal Incidence

For guided mode resonances (GMRs) excited at normal incidence, the in-plane wavevector vanishes and the excited mode is a superposition of forward and backward propagating waves with equal amplitude. The transverse field profiles are either even or odd with respect to the center of the unit cell. For transverse electric (TE) and transverse magnetic (TM) modes, the relevant field components exhibit well-defined parity. In the case of TE modes, which contain a single electric field component, this component is either even or odd. Meanwhile, TM modes contain one even and one odd component.

The nonlinear overlap integral then depends on the product of these field components. In the case of nonlinear interactions governed by the tensor elements $\chi^{(2)}_{xzx}, \chi^{(2)}_{xxz}, \chi^{(2)}_{yzy}, \chi^{(2)}_{yyz}$, one requires a TM mode to enable the two orthogonal field components. Because E_x and E_z have opposite parity in symmetric TM modes, their product is odd, and the volume integral vanishes for coupling to a plane-wave second harmonic field. As a result, the modal overlap, β, vanishes at normal incidence, and the corresponding nonlinear process is symmetry-forbidden despite the presence of non-zero local fields.

This symmetry constraint is specific to tensor elements involving orthogonal field components. For co-polarized interactions (e.g., $\chi^{(2)}_{zzz}$), the integrand remains even, and the overlap is non-zero. This explains why GMRs can efficiently enhance second harmonic generation in materials such as $LiNbO_3$, while the interband-MQW tensor elements considered here remain inaccessible under symmetric excitation conditions.

2.2 Symmetry Breaking

A nonzero modal overlap can be achieved by breaking the symmetry of the guided mode. This can be accomplished, for instance, by using a finite angle of incidence or by introducing structural asymmetry into the metasurface unit cell. In both cases, the parity of the field components is modified, leading to a nonzero integrand in the modal overlap integral and enabling efficient second harmonic generation.

This behavior is observed in both the simulations and experiments presented in the main text, where a small angle of incidence leads to a rapid increase in the modal overlap and nonlinear signal.

3 Second Harmonic Generation Conversion Efficiency

The second harmonic signal is measured measured using an Andor SR-500i-B2-R spectrometer with an Andor Newton 971 EMCCD detector, which records digital counts. For conversion to photon flux, we calibrate the digital count with the electron multiplication gain, quantum efficiency, and sensitivity of the detector.

The resulting photon flux is corrected for optical losses in the detection path, including mirror reflectivity, diffraction efficiency of the spectrometer and fiber efficiency. The second harmonic power is then given by $P_{SH} = N_{photons} \cdot \hbar\omega_{SH}$.

To estimate conversion efficiency, the second harmonic power is normalized to the incident pump intensity. The pump intensity is calculated from the measured average power, P_{in}, assuming a Gaussian beam profile,

$$I_{pump} = \frac{2P_{in}}{f_{rep}\tau A}, \tag{3}$$

where f_{rep} is the repetition rate of the laser, τ is the pulse duration, and A is the beam area. A correction factor is applied to account for the fraction of the incident power coupled into the resonant mode, following the procedure outlined in the Methods.

Using this intensity, we define a normalized second harmonic conversion efficiency as

$$\eta_{norm} = \frac{\eta}{I_{pump}} = \frac{P_{SH}}{P_{in} \cdot I_{pump}}. \tag{4}$$

We obtain a normalized second harmonic generation conversion efficiency of $\eta_{normalized} \approx 1.4 \cdot 10^{-4}$ $\mathrm{cm^2GW^{-1}}$, averaged over pump powers from 30 mW to 80 mW. We note that we do not observe photodamage for the powers used for this estimate. This estimate depends on assumptions regarding the beam profile, pulse duration, effective interaction area, and coupling efficiency into the resonant mode, and therefore carries a systematic uncertainty not present in the susceptibility extraction used in the main text. We note that the value should be regarded as conservative, as the experimental configuration was optimized for relative calibration of $\chi^{(2)}$ against the $LiNbO_3$ reference rather than for maximizing absolute second harmonic collection efficiency.

4 Supplementary Figures

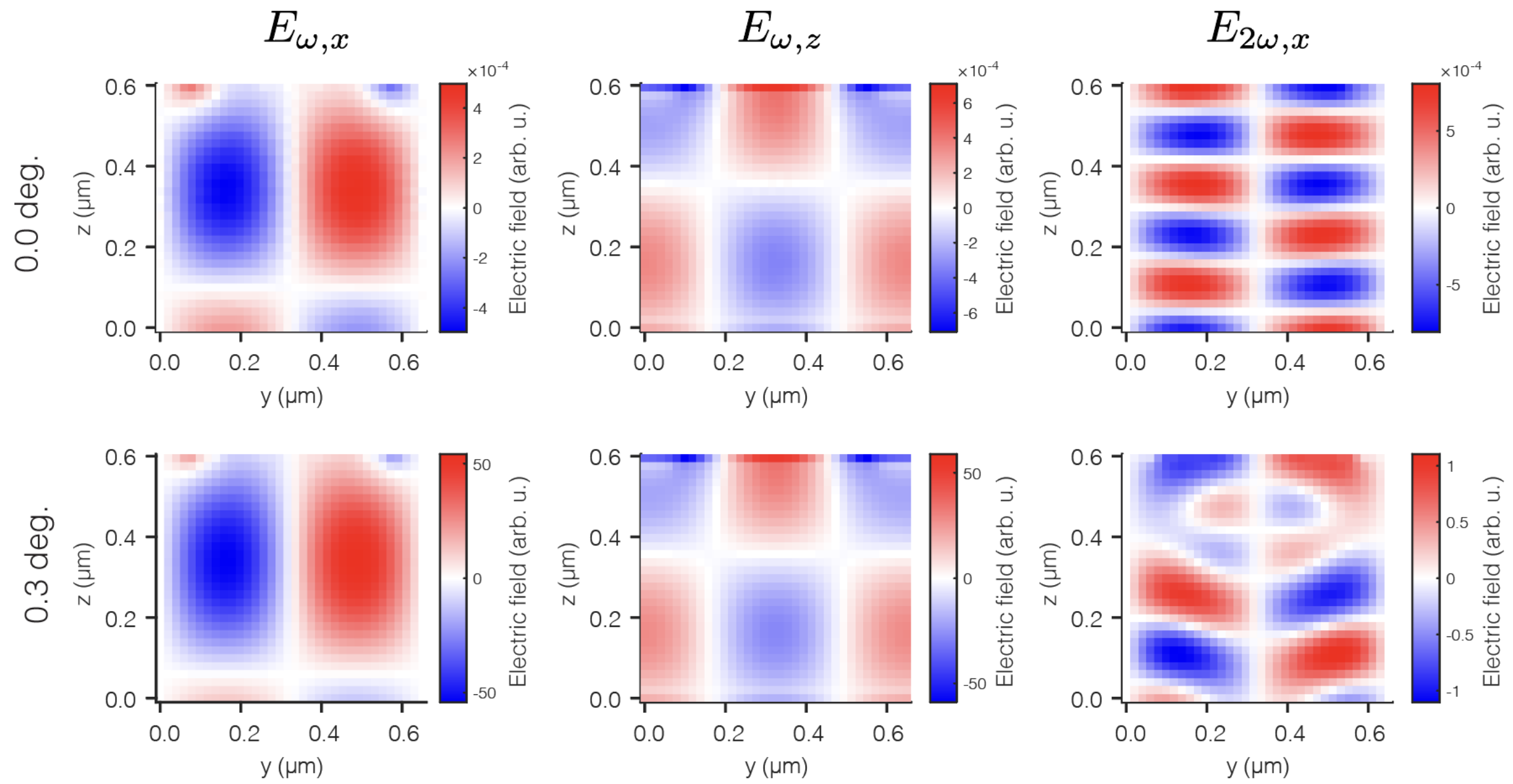

Figure S1. Field distribution in the waveguide at resonance. The panels show the spatial (y-z) profile of the simulated fields (arb. units) in the waveguide, summed along the x-direction of the unit cell. We show the x and z component of the pump field and the x component of the second harmonic field for normal and 0.3° incidence angle for the left branch of resonance B.

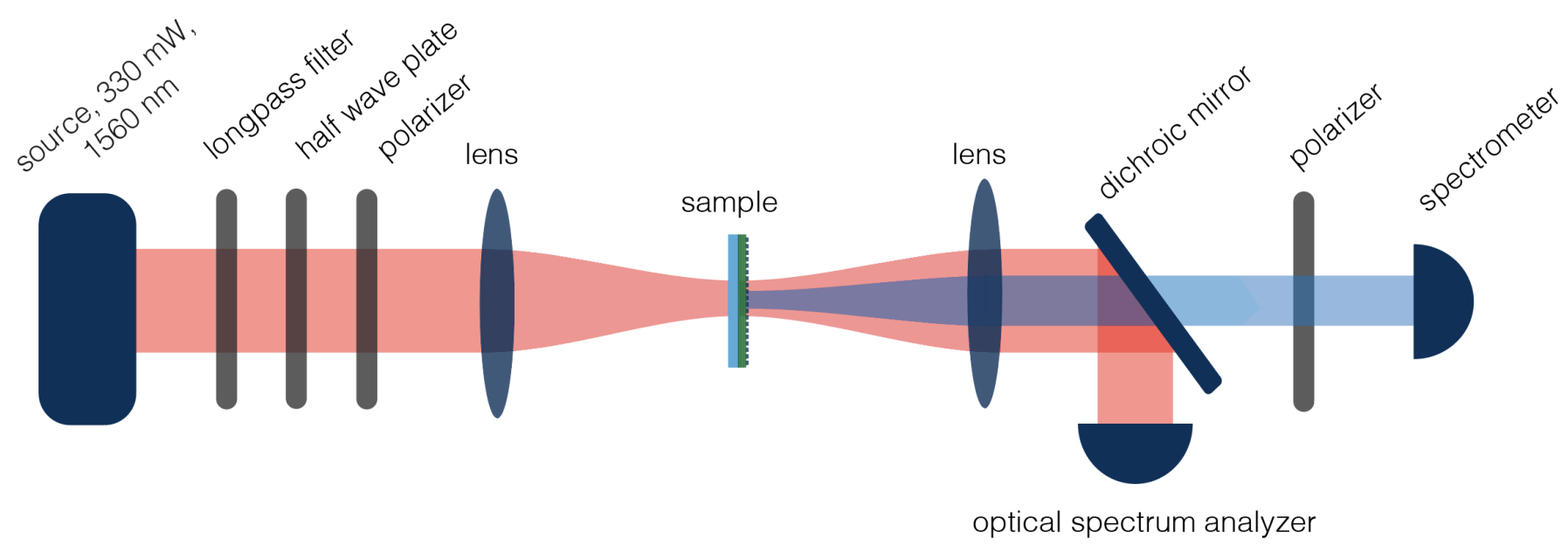

Figure S2. Schematic of the optical setup for device characterization. 70 fs pulsed light from a MenloSystems ELMO Femtosecond Erbium laser passes through a long pass filter to remove second harmonic light coming from the laser, followed by a half-waveplate and a polarizer for polarization control. A low numerical aperture lens with a focal length of 40 cm focuses light onto the sample. The light is collected by a 50 cm focal length lens, followed by a dichroic mirror to block the pump frequency. A second polarizer enables measurements of the output polarization, and the output is detected using an Andor SR-500i-B2-R spectrometer. The pump frequency is collected using an Anritsu MA9710B optical spectrum analyzer.

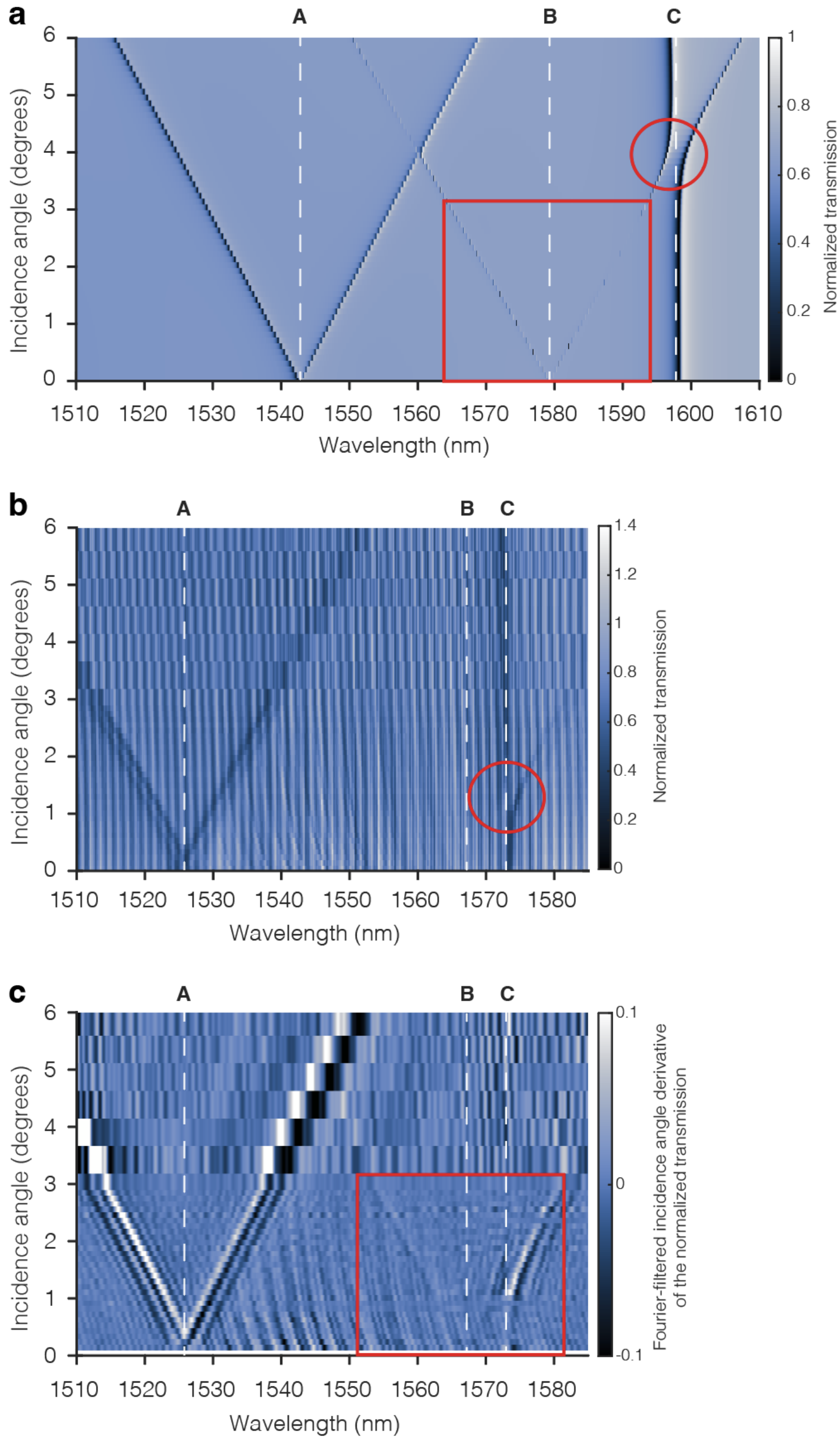

Figure S3. Incidence angle-resolved transmission spectra of the metasurface for x-polarized illumination. **a** Simulated results. **b** Experimental measurements. The high quality factor resonance B is most visible in the anti-crossing with resonance C, marked by a red circle in panels **a** and **b**. Note that a relative wavelength shift between resonances B and C leads to the anti-crossing occurring at a smaller angle in the experimentally measured spectra compared to the simulations. **c** Fourier-filtered incidence angle derivative of the experimental measurements. Here, resonance B is visible, marked by a red square in panels **a** and **c**.

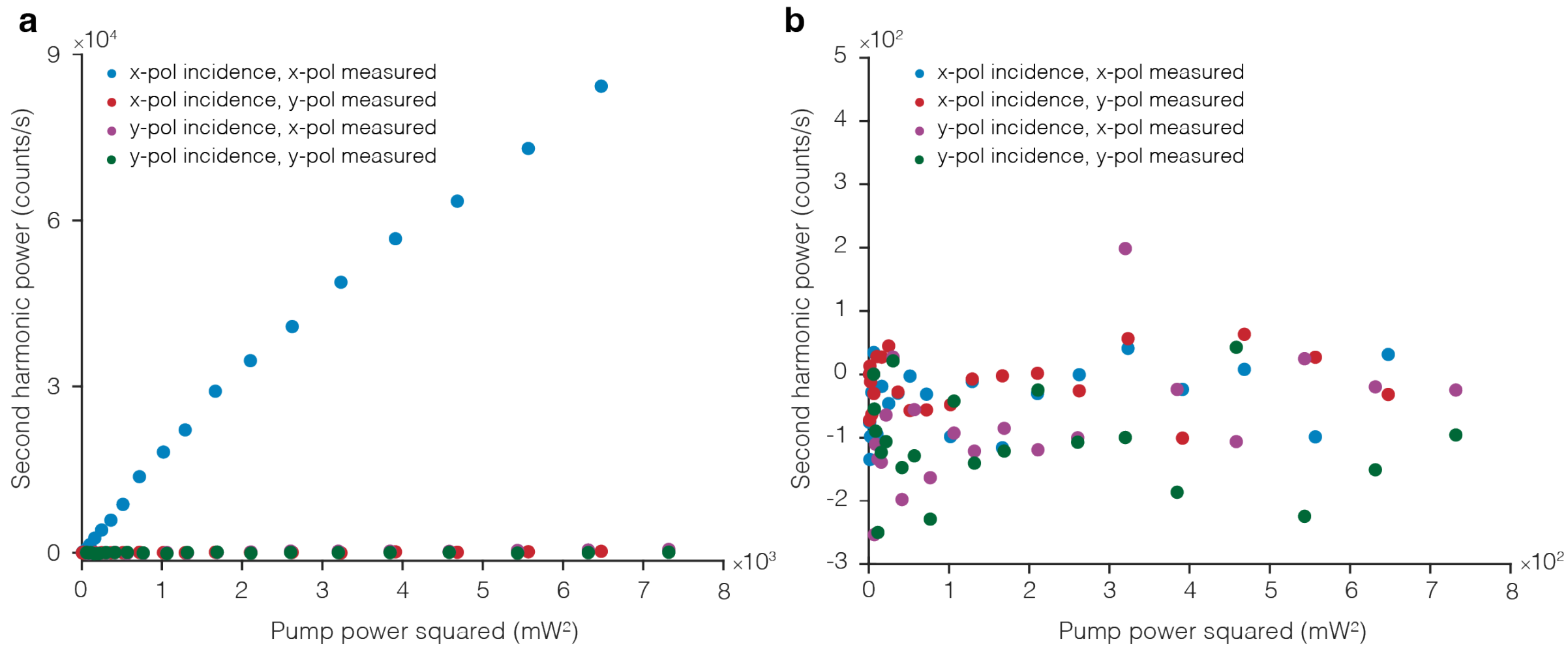

Figure S4. Nonlinear response of the metasurface/heterostructure sample and the bare heterostructure. Measured generated second harmonic power as a function of the squared average incident pump power and the input and output polarization (linear x and y polarized light) for **a** the metasurface/heterostructure sample, and **b** the bare heterostructure, both measured at 0.3° angle of incidence.

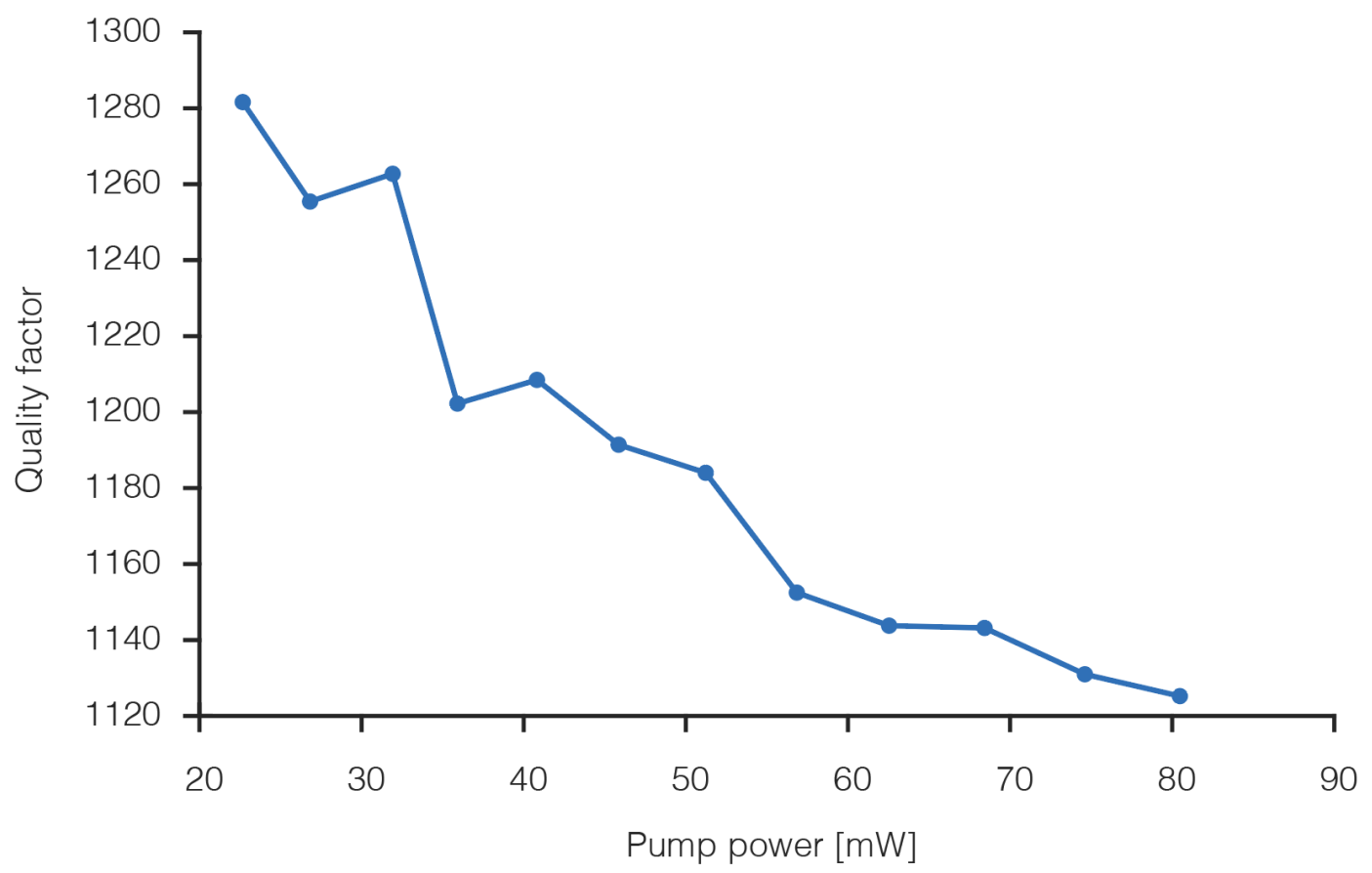

Figure S5. Pump-power-dependent quality factor of the resonance. The quality factor at the pump wavelength is extracted from a squared Lorentzian fit to the second harmonic response.

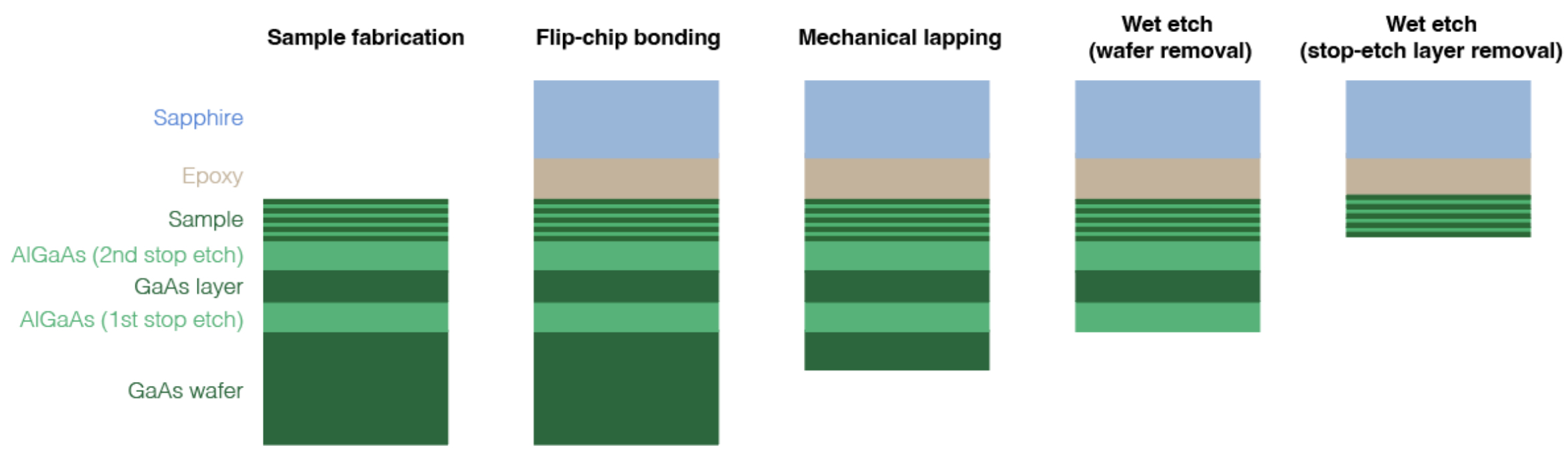

Figure S6. Substrate transfer. The sample is grown via molecular beam epitaxy and subsequently flip-chip bonded to a 500-μm thick sapphire substrate using 353ND EPO-TEK epoxy resin. Mechanical lapping is used for the first stage of the GaAs substrate removal, followed by a wet etch removal of the remaining GaAs substrate and the etch-stop layers.

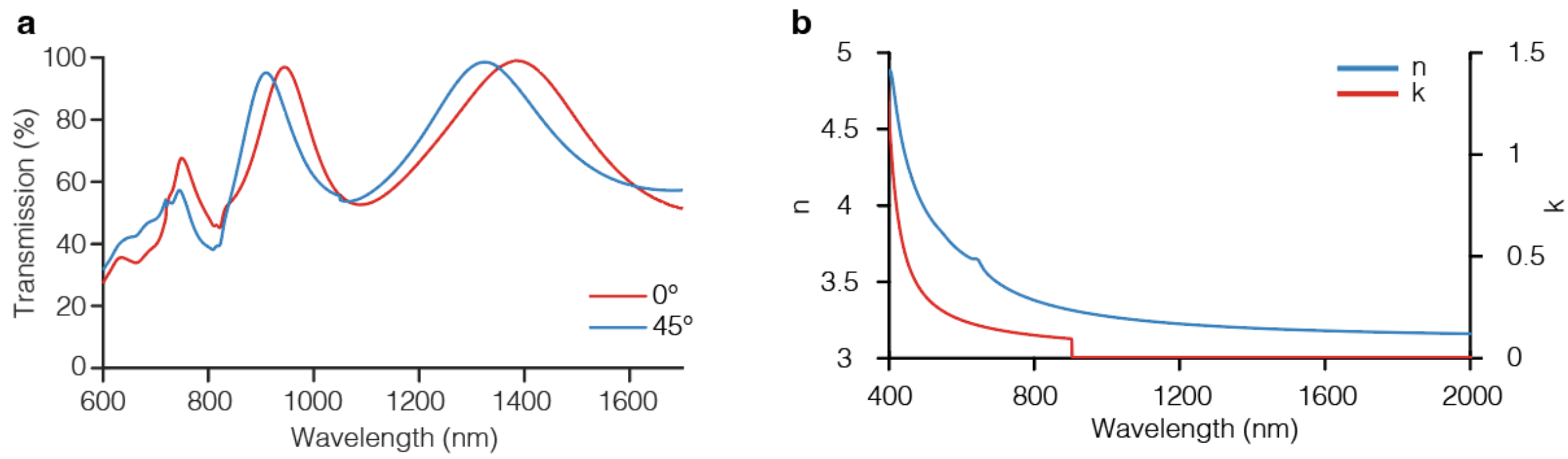

Figure S7. Characterization of the substrate transferred heterostructure. a Transmission spectrum of a 16-period MQW film on sapphire measured at incidence angles of 0° and 45° using a Cary 7000 UV-Vis-NIR Universal Measurement Spectrophotometer. Linear transmission measurements of the grown sample at normal and 45° incidence show only above-band gap absorption and thin-film Fabry Perot interference. Grating change at 720 nm, detector change at 1050 nm. **b** Refractive index measurements of the heterostructure on a sapphire substrate taken using a Woollam RC2 Spectroscopic Ellipsometer.

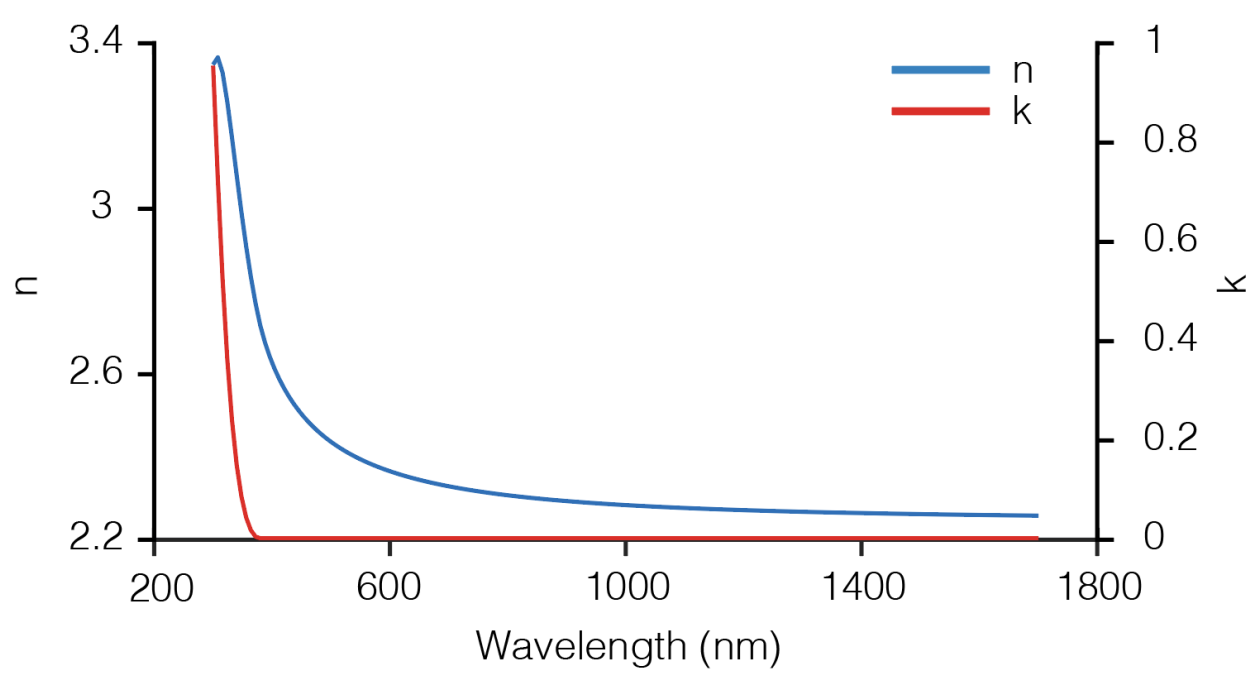

Figure S8. Characterization of TiO_2. Refractive index of an atomic-layer-deposited TiO_2 thin film on a Si substrate, measured by spectroscopic ellipsometry (Woollam RC2).

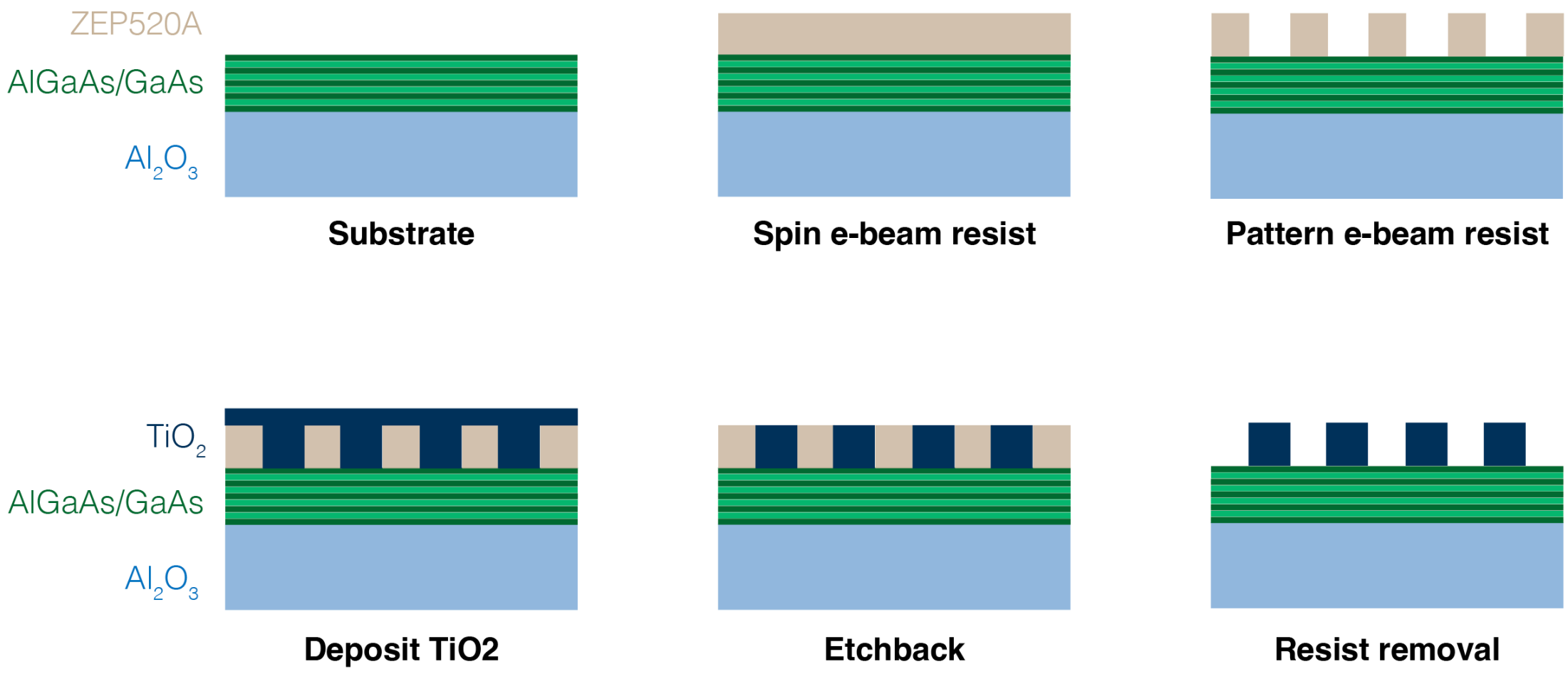

Figure S9. Metasurface fabrication. Positive-tone E-Beam resist is spin coated on the surface of the heterostructure and exposed using electron beam lithography. The exposed resist is removed using developer and the gaps are filled with TiO_2 by atomic layer deposition. The overgrown TiO_2 is removed by reactive ion etching, and the remaining resist is removed.

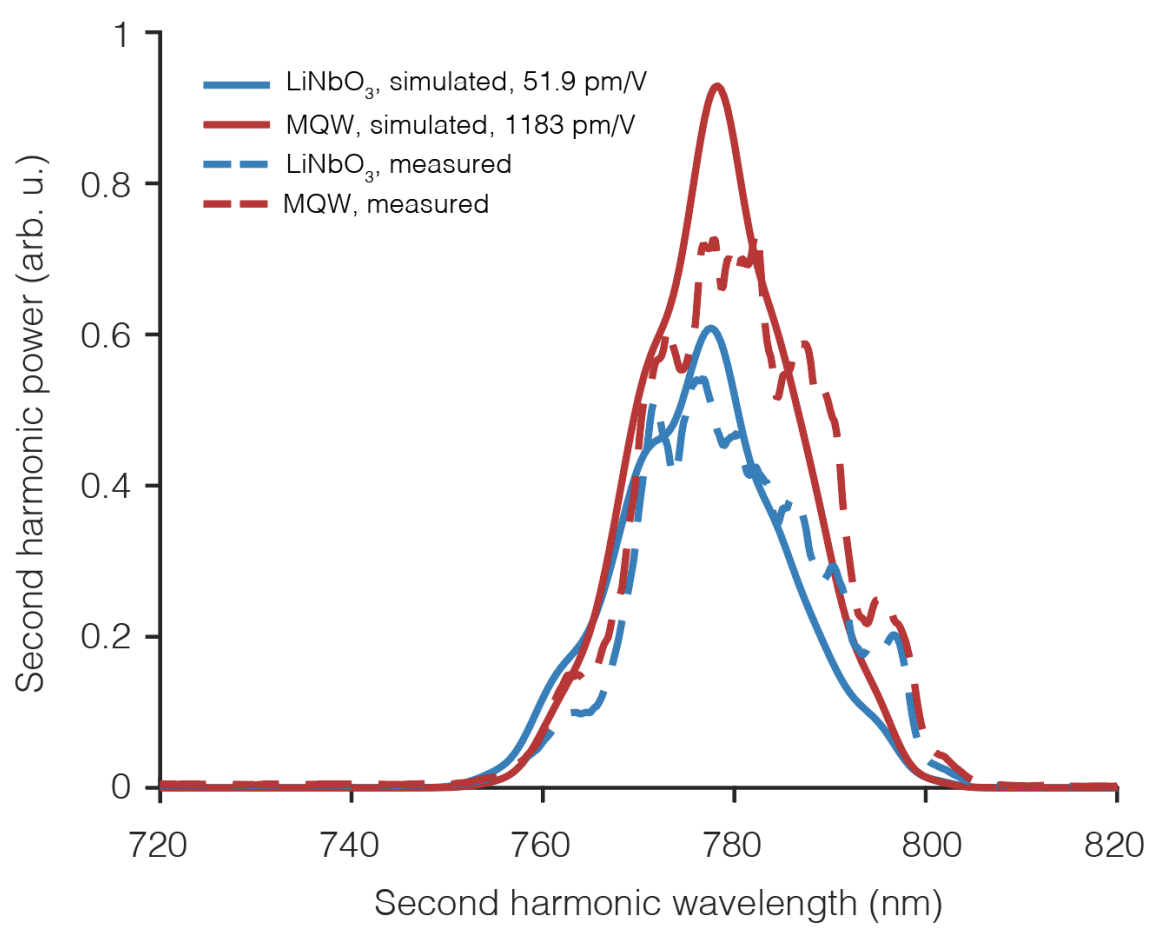

Figure S10. Measured and modeled sum-frequency spectra. The output sum-frequency spectra of a 16-period MQW on sapphire measured at 45° incidence and x-cut $LiNbO_3$ measured at 0° incidence under x-polarized illumination. Dashed lines indicate experimental measurements, while solid lines show simulated sum-frequency spectra derived from the measured incident spectrum. Both blue and red curves yield the same integrated power, respectively.
