# Supplementary material for: Quantum-well metasurface for free-space-accessible enhanced nonlinear polarization
Source: Nat Nanotechnol. 2026 Sep 2;21(11):1558–64. doi: 10.1038/s41565-026-02268-0 (PMC13754578; doi:10.1038/s41565-026-02268-0)
Supplement: Supplementary file 2 [file 41565_2026_2268_MOESM2_ESM.pdf]

nature portfolio

Peer Review File

Quantum-Well-Metasurface for Free-Space-Accessible Enhanced Nonlinear Polarization

Corresponding Author: Ms Pernille Undrum Fathi

This file contains all reviewer reports in order by version, followed by all author rebuttals in order by version.

A version of this paper was originally rejected for publication by Nature Nanotechnology, however that decision was reconsidered after appeal by the authors.

Version 0:

Reviewer comments:

Reviewer #1

(Comments for the Author)
In this work, the authors combine a bandstructure engineered GaAs/AlGaAs heterostructure with a high Q-factor dielectric metasurface to tailor both the intrinsic nonlinear susceptibility and the light field localization within the heterostructure. They realize a second-order nonlinear tensor element with value up to 1.6 nm/V at 1.57 µm wavelength. They also show that the effective nonlinear tensor element with value of 14 nm/V can be obtained using a TiO_2 metasurface fabricated on the heterostructure. Both the theoretical and the experimental results are well presented. The proposed strategy shows a scalable route to realize highly efficient nonlinear nanophotonic devices. I would like to support the publication of this work in Nature Nano after addressing the following comments and suggestions.

Comments:
1. In Figure 3a, it is better to plot the crystalline structure of the quantum well, for example, if the quantum well has rotational symmetry along z-axis or symmetry breaking property with respect to y-axis. Otherwise, it is difficult to understand why x-direction is important.
2. It is discussed that without metasuface the quantum well device has similar SHG efficiency to that of the LN control sample. How about the feasibility to design a LN-metasurface device by using the strategy in this work? Compared to the quantum well platform in this work, if the LN thin film based device is better or not, considering the difficulty of materials growth, working bandwidth and nonlinear efficiency.
3. When calibrating the effective nonlinear susceptibility of the hybrid device, many textbook methods assume that the pump laser is a continuous wave. As the pumping is from a femtosecond laser, it is important of clarify if the authors consider the peak power or just averaged power to evaluate chi(2).

Reviewer #2

(Comments for the Author)
I have read the manuscript by Undrum Fathi et al. I particularly appreciated the transparency of the authors adding Ref. 38, which is available in an open access database and in co-authored by some of the authors of the present paper. I would like first to point out that the manuscript reports an exceptionally large effective second-order nonlinear susceptibility (~14 nm/V), which can be considered, at least in the NIR range of the spectrum and to the best of my knowledge, among the highest values demonstrated to date. This level of performance is certainly very relevant and represents a significant technical achievement that will be of clear interest to the nonlinear photonics community. However, I find that there are some crucial points which prevent the manuscript from being suitable for publication in Nature Nanotechnology.
A central concern regarding the level of novelty is that the manuscript extends, rather than fundamentally departing from, the conceptual framework already established in Ref. [38]. While the reported nonlinear properties are remarkable in absolute, the authors themself point out that it is approximately one order of magnitude compared to Ref. [38], suggesting a quantitative improvement rather than a qualitative breakthrough. In particular, the authors emphasize that metasurfaces enable access to “otherwise unusable nonlinear tensor elements”; however, this capability has already been convincingly demonstrated in the nonlinear metasurface literature. For example, lithium niobate metasurfaces incorporating multiple, differently oriented metagratings have shown that the orientation of the nanostructure can directly control the polarization

and nonlinear emission pathways, effectively selecting different tensor components either by patterning directly the material or in an etchless configuration [e.g. ACS Photonics 2021 8 (3), 731-737 DOI: 10.1021/acsphotonics.1c00026; Nano Lett. 23, 8091-8098 (2023) DOI: 10.1021/acs.nanolett.3c02055]. In particular, the use of GMR to enhance nonlinear optical processes is to date well established [e.g. Phys. Rev. B 106, 125411 (2022) DOI: https://doi.org/10.1103/PhysRevB.106.125411; Phys. Rev. Lett. 127, 153901 (2021) DOI: https://doi.org/10.1103/PhysRevLett.127.153901]. The authors are also among the main contributors in this field in the past decade. For instance, lithium niobate metasurfaces and related platforms have shown that careful engineering of resonant modes and polarization fields allows selective enhancement and probing of different nonlinear tensor components, as well as efficient in- and out-coupling of light. In this context, I am afraid that the use of metasurfaces to both enhance coupling and access specific tensor elements cannot be regarded as conceptually new.
Let me also add that the authors here focus more on the physics, estimating the nonlinear susceptibility. However, by looking at Figure 2f, it seems the absolute signals are not far off from the $LiNbO_3$ thin film. How thick is the film they compared the MQW to? Looking at the numbers, I assume it is much thicker than the MQW sample. Here another question arises. In literature it is possible to find transition metal dichalcogenide (TMDs) materials with extremely large (at least claimed) susceptibility. Yet, given the small volumes involved the absolute conversion efficiency remains quite low before arriving to photodamage. Since this paper is clearly meant for a nanotechnology audience I believe the absolute conversion efficiency achievable in terms of P_{SHG}/P_{in} and, more crucially, conversion efficiency normalized to the input intensity should be given. I would indeed expect to see in both Figure 2f and 4b,c on the vertical axis SHG powers in W to be able to compute the conversion. From a technological point of view, I believe this is extremely relevant.
Something related to the maximum conversion efficiency is the possible absorption mechanisms. The change in slope in Figure 4b is attributed by the authors to a two-photon absorption process that competes with SHG. In particular, the fact that the system is based on interband SHG mechanism, where presumably resonant states are involved, could be a crucial limiting factor. I am wondering if this might result in a lower level of photodamage threshold with respect to nonlinear processes in transparent materials (between non-resonant levels).
Besides the above comments, which have to considered and could improve putting the paper into the right context, I believe that the primary advance of the present work lies in the photonic optimization and the performance enhancement of the structure devised in Ref [38], rather than in a fundamentally new nanotechnological concept. While the results are clearly sound and remarkable from a nonlinear optics perspective and the paper well-written, the nanotechnological innovation—particularly in terms of heterostructure design and platform development—is limited here and was arguably more central in Ref. [38]. This distinction is crucial given the scope of Nature Nanotechnology, which emphasizes conceptual advances in nanotechnology (of course in addition to performance metrics). In this context, and considering that a group of authors—presumably those involved in the quantum well fabrication—contributed to both studies, the present manuscript reads as a natural continuation and refinement of the same platform. This raises the possibility that an integration of the two works could more clearly establish the level of conceptual advancement deserving consideration in Nature Nanotechnology. Conversely, as it stands, the current manuscript alone appears to represent an incremental step beyond prior results.

Reviewer #3

(Comments for the Author)
In this manuscript, the authors report enhanced second-harmonic generation (SHG) based on a combination of GaAs/AlGaAs multi-quantum-well (MQW) heterostructures and a metasurface supporting guided-mode resonances in the infrared. This field has been heavily studied for the past 10 years, including many publications by Igal Brener's group, a co-author of this manuscript. In this manuscript, by tuning the interband transition in the MQW heterostructures, the nonlinear coefficients $\chi_{xzx}^{(2)}$, $\chi_{xxz}^{(2)}$, $\chi_{yzy}^{(2)}$, $\chi_{yyz}^{(2)}$, were enhanced. Combined with the resonantly enhanced electric field in the z-direction induced by the guided-mode resonance, this enhancement leads to a pronounced enhancement of SHG. In addition, the manuscript presents the angular dispersion of both the linear and nonlinear responses of the designed metasurface. Although this approach is somehow new, the novelty is hardly sufficient for such a high-impact journal. This manuscript is better suited to journals such as Nano Letters or Nature Communications.

Nevertheless, my detailed comments and concerns are outlined below.

- The authors use an x-cut $LiNbO_3$ thin film as a reference to demonstrate the nonlinearity of the reported MQW heterostructures. Although $LiNbO_3$ may exhibit a comparable $\chi_{zzz}^{(2)}$ coefficient, its second-order nonlinear tensor is different from that of GaAs/AlGaAs. Other tensor components may also contribute to the observed response. Why did the authors not use a GaAs/AlGaAs thin film as a more appropriate reference for comparison?
- The refractive index is a crucial parameter in the design of guided-mode resonances, particularly for predicting their Q-factors and angular dispersion. Could the authors provide the effective refractive indices of the MQW heterostructures and TiO_2?
- In Fig. 3(b), only the electric-field distributions in the xy plane are presented. Since the field enhancement associated with guided-mode resonances is typically non-uniform across the thickness of the film, especially within the MQW heterostructures, could the authors also provide the electric-field distributions in the xz or yz plane? Could the authors clarify how the field distribution across the thickness of the film would affect the SHG enhancement?
- In Fig. 3(f), the overlap factor (β) appears to remain very small for resonance C, suggesting that the electric field in the z-direction is not enhanced. Could the authors clarify the reason for this?
- Why do the overlap factor (β) and the SHG intensity both reach their maximum at an incident angle of 0.3°? Is this primarily related to the Q-factor, the field distribution, or another mechanism? A clear and detailed explanation is needed, particularly given the substantial variation in Q-factor. At present, it is still unclear why the SHG emission differs so markedly between incident angles of 0.3° and 1°.
- Since the in-plane symmetry is broken when the incident angle is varied, the authors should specify the direction along which the incident angle is tuned, that is, whether the change is introduced through the p_x or p_y momentum component.

Version 1:

Reviewer comments:

Reviewer #1

(Comments for the Author)
The authors have sucessfully addressed my previous comments and suggestions, I would like to support the publication of this work in Nature Nnaotechnology.

Reviewer #2

(Comments for the Author)
I have read the authors' reply. All technical issues have been adequately addressed. However, I remain concerned about double publication.
The reported effective susceptibility is a record to date, especially at telecom wavelength, and deserves visibility in the literature. But the more significant result is the one already published in reference 41, which introduces the genuine novelty: boosting the nonlinearity through interband engineering. From a nanotechnology standpoint, that achievement is the striking one; the metasurface engineering in the present work is comparatively standard.
It is true that this paper does unlock the device's full potential by breaking the symmetry and accessing the stronger nonlinear tensor element, but this step is not groundbreaking from a nanophotonics perspective. The speculative new applications this work and results threin might enable could certainly raise the interest of the nanophotonic community, but I do not think this is sufficient to warrant publication in Nature Nanotechnology.

Reviewer #3

(Comments for the Author)
The authors have responded well to my concerns. I am now in a position to recommend this manuscript for publication in Nature Nanotechnology.

Open Access This Peer Review File is licensed under a Creative Commons Attribution 4.0 International License, which permits use, sharing, adaptation, distribution and reproduction in any medium or format, as long as you give appropriate credit to the original author(s) and the source, provide a link to the Creative Commons license, and indicate if changes were made.
In cases where reviewers are anonymous, credit should be given to 'Anonymous Referee' and the source.
The images or other third party material in this Peer Review File are included in the article’s Creative Commons license, unless indicated otherwise in a credit line to the material. If material is not included in the article’s Creative Commons license and your intended use is not permitted by statutory regulation or exceeds the permitted use, you will need to obtain

permission directly from the copyright holder.
To view a copy of this license, visit https://creativecommons.org/licenses/by/4.0/

Answers to the reviewers

Below, we provide a point-by-point response (printed in blue) to the reviewer's comments (black) and include changes to the manuscript in green.

REVIEWER COMMENTS

Reviewer #1 (Comments for the Author):

In this work, the authors combine a bandstructure engineered GaAs/AlGaAs heterostructure with a high Q-factor dielectric metasurface to tailor both the intrinsic nonlinear susceptibility and the light field localization within the heterostructure. They realize a second-order nonlinear tensor element with value up to 1.6 nm/V at 1.57 μm wavelength. They also show that the effective nonlinear tensor element with value of 14 nm/V can be obtained using a TiO_2 metasurface fabricated on the heterostructure. Both the theoretical and the experimental results are well presented. The proposed strategy shows a scalable route to realize highly efficient nonlinear nanophotonic devices. I would like to support the publication of this work in Nature Nano after addressing the following comments and suggestions.

Response: We thank Reviewer #1 for their positive evaluation of our work and supporting publication in *Nature Nanotechnology*.

Comment: In Figure 3a, it is better to plot the crystalline structure of the quantum well, for example, if the quantum well has rotational symmetry along z-axis or symmetry breaking property with respect to y-axis. Otherwise, it is difficult to understand why x-direction is important.

Response 1.1: We thank the reviewer for pointing this out and agree that the manuscript did not clearly explain why the x-direction was chosen.

The choice of x versus y is arbitrary at the material level. As stated in the manuscript, selection rules for the MQW-interband transition yield the dominant tensor elements $\chi^{(2)}_{xzx} = \chi^{(2)}_{xxz} = -\chi^{(2)}_{yzy} = -\chi^{(2)}_{yyz}$ with equal magnitude. The MQW inherits the in-plane symmetry of GaAs and AlGaAs, which both have zincblende structure (point group $\underline{4}3m$). While the heterostructure breaks the symmetry along the growth direction z ([001]), the in-plane directions x ([100]) and y ([010]) remain equivalent.

In our device, we excite a guided mode resonance (GMR) that couples to the x-based tensor elements. An equivalent response could be obtained by rotating the metasurface unit cell by 90° and exciting the corresponding y-based tensor elements.

Given the large size of our MQW structure, a full plot of its crystal structure is visually complex. To clarify the manuscript, we now describe the crystal structure in the text, and clarify that the choice of the x direction yields no loss of generality:

The MQW inherits the in-plane symmetry of the zincblende GaAs/AlGaAs crystal structure (point group $\underline{4}3m$). While the heterostructure breaks symmetry along the growth direction z ([001]), the in-plane directions x ([100]) and y ([010]) remain physically equivalent at the material level.

We illuminate the heterostructure with x-polarized, 70-fs long laser pulses (see the Supplementary Information) to determine its $|\chi^{(2)}_{xzx} + \chi^{(2)}_{xxz}|$ in an optical measurement. The choice of probing the x-based tensor elements is arbitrary, and an equivalent response would be obtained for the corresponding y-based tensor elements.

Comment: It is discussed that without metasuface the quantum well device has similar SHG efficiency to that of the LN control sample. How about the feasibility to design a LN-metasurface device by using the strategy in this work? Compared to the quantum well platform in this work, if the LN thin film based device is better or not, considering the difficulty of materials growth, working bandwidth and nonlinear efficiency.

Response 1.2: Metasurface-based enhancement has been applied to lithium niobate ($LiNbO_3$) successfully. We have included the following references to convey this point:

Carletti, L., Zilli, A., Moia, F., Toma, A., Finazzi, M., De Angelis, C., Neshev, D. N. & Celebrano, M. *Steering and Encoding the Polarization of the Second Harmonic in the Visible with a Monolithic $LiNbO_3$ Metasurface*. ACS Photonics 8, 731–737. doi:10.1021/acsphotonics.1c00026 (2021).

Zhao, Y., Chen, Z., Wang, C., Yang, Y. & Sun, H. -B. *Efficient second- and higher-order harmonic generation from $LiNbO_3$ metasurfaces.* Nanoscale 15, 12926–12932. doi:10.1039/D3NR02430J (2023)

Zheng, Z., Xu, L., Huang, L., Smirnova, D., Hong, P., Ying, C. & Rahmani, M. *Boosting second-harmonic generation in the $LiNbO_3$ metasurface using high-Q guided resonances and bound states in the continuum*. Physical Review B 106, 125411. doi:10.1103/PhysRevB.106.125411 (2022)

A key difference between $LiNbO_3$ and the interband MQW platform presented here lies in the nonlinear tensor elements being accessed. In $LiNbO_3$, the dominant tensor element, $\chi^{(2)}_{zzz}$, is driven by co-polarized pump photons and is therefore directly accessible with GMRs. In contrast, the interband MQW tensor elements require orthogonal field components, which necessitates transverse-magnetic guided modes. Here the field components exhibit opposite symmetry, leading to vanishing modal overlap unless the symmetry is broken.

However, the final performance is given by the effective nonlinear response of a resonant device, which scales with the intrinsic $\chi^{(2)}$, the resonant field enhancement, and the modal overlap. The MQW platform exhibits an intrinsic nonlinearity that is two orders of magnitude larger than $LiNbO_3$, enabling a strong nonlinear response without requiring extremely high-Q resonances. In $LiNbO_3$-based systems, achieving comparable performance would require considerably higher Q-factors. However, in free-space metasurfaces, compensation through an increased Q factor alone is limited by scattering, finite-size effects, and fabrication imperfections. Even if successful, a high Q factor decreases the bandwidth of the device. The combination of MQW materials with metasurfaces, therefore, enables broadband devices with comparable efficiencies to narrowband $LiNbO_3$ counterparts.

We now summarize this in the manuscript:

> High-quality resonant metasurfaces for nonlinear enhancement have previously been demonstrated in other materials such as $LiNbO_3$. Still, the nonlinear susceptibilities of the underlying material platforms fundamentally limit their performance. While these devices can compensate for lower nonlinearity through higher quality factors, this introduces a trade-off with the operational bandwidth and practical fabrication constraints.

Comment: When calibrating the effective nonlinear susceptibility of the hybrid device, many textbook methods assume that the pump laser is a continuous wave. As the pumping is from a femtosecond laser, it is important of clarify if the authors consider the peak power or just averaged power to evaluate chi(2).

Response 1.3: Our extraction of the effective nonlinear susceptibility does not assume a continuous-wave. Instead, our simulations use the time-dependent electric pump field E(t), thereby accounting for the femtosecond nature of the pump pulses. The nonlinear polarization is computed directly in the time domain as $P^{(2)}(t) \propto \chi^{(2)} E^2(t)$. Importantly, the calibration is performed relative to a $LiNbO_3$ reference measured under identical conditions. As a result, the absolute field amplitude cancels, and the extracted $\chi^{(2)}$ does not depend on whether the pump is expressed in terms of peak power or average power.

We clarify this in the Methods by including the following:

> The use of a time-dependent electric field $E(t)$ inherently accounts for the pulsed nature of the excitation and avoids the need for a continuous-wave approximation. In particular, the nonlinear polarization is computed as $P(t) \propto \chi^{(2)} E^2(t)$, such that the temporal profile of the pulse, including its peak intensity, is explicitly captured in the simulation. As the extraction of $\chi^{(2)}$ is performed through a relative calibration against a $LiNbO_3$ reference measured under identical excitation conditions, the absolute amplitude of the electric field cancels. Consequently, the extracted $\chi^{(2)}$ does not depend on whether the pump is expressed in terms of peak power or average power.

Reviewer #2 (Comments for the Author):

Comment: I have read the manuscript by Undrum Fathi et al. I particularly appreciated the transparency of the authors adding Ref. 38, which is available in an open access database and in co-authored by some of the authors of the present paper. I would like first to point out that the manuscript reports an exceptionally large effective second-order nonlinear susceptibility (~14 nm/V), which can be considered, at least in the NIR range of the spectrum and to the best of my knowledge, among the highest values demonstrated to date. This level of performance is certainly very relevant and represents a significant technical achievement that will be of clear interest to the nonlinear photonics community.

We thank Reviewer #2 for the positive assessment of our work.

Comment: However, I find that there are some crucial points which prevent the manuscript from being suitable for publication in Nature Nanotechnology.
A central concern regarding the level of novelty is that the manuscript extends, rather than fundamentally departing from, the conceptual framework already established in Ref. [38]. While the reported nonlinear properties are remarkable in absolute, the authors themself point out that it is approximately one order of magnitude compared to Ref. [38], suggesting a quantitative improvement rather than a qualitative breakthrough.

Response 2.1: We appreciate the opportunity to clarify the advance outlined in our manuscript and distinguish between the present work and Ref. 38.

We note that Ref. 38 was made publicly available on the arXiv following an editorial request during the review process, because we included a reference to it. It was not part of the established literature at the time of submission. Importantly, Ref. 38 focuses on the material design, optimization, and growth of the heterostructure, primarily directed toward a materials science audience. The present manuscript explains the heterostructure's working principle and enhancement mechanism for a broad audience and demonstrates its applicability using a device-level concept that enables its practical use and then benchmarks it against the current workhorse of integrated nonlinear optics.

As demonstrated by this benchmark, the immense interband-engineered second-order susceptibilities in Ref. 38 cannot be used in free-space settings and require nanophotonic engineering to extract their nonlinear response.

To clarify this in the manuscript, we now emphasise that two different observables are relevant: In the MQW film, we extract the intrinsic material nonlinearity, which we renamed ${\chi^{(2)}}_{intrinsic,MQW}$. The intrinsic susceptibility, $\chi^{(2)}_{intrinsic,MQW} = |\chi^{(2)}_{xzx} + \chi^{(2)}_{xxz}| \approx 1.6\, nm/V$, is consistent with the values reported in Ref. 38 (note that the latter uses a MQW-literature specific assumption that sets $\chi^{(2)}_{xxz} = 0$, which we avoid here to keep the manuscript accessible to the general nonlinear optics community). The intrinsic susceptibility does not capture whether a tensor element is usable. Meanwhile, for the metasurface-MQW device, we define a free-space effective

nonlinearity, $\chi^{(2)}_{eff,MQW+MS} = \chi^{(2)}_{xxx,MQW+MS} \approx 14\ nm/V$, that characterizes the optically accessible nonlinear response, and also captures eventual absorption losses and device enhancement effects.

Figure 2f of our manuscript, where the unpatterned MQW film produces a second harmonic flux comparable to that of a $LiNbO_3$ film of equal thickness, highlights the importance of the metasurface-MQW combination (see also response 2.2). We now stress this fact in the manuscript:

> While the GaAs/AlGaAs heterostructure exhibits a large $\chi^{(2)}_{intrinsic,MQW}$, this response remains inefficient under conventional free-space excitation, resulting in second harmonic generation comparable to established materials such as $LiNbO_3$. By introducing symmetry-broken, resonantly enhanced electromagnetic fields, the metasurface enables efficient coupling to these intrinsic nonlinearities and provides an additional enhancement of the nonlinear response. This results in a transition from a material with large but underutilized nonlinear properties to a functional nanophotonic platform with strongly enhanced effective nonlinearity. Even larger nonlinear responses can be achieved by further optimizing fabrication and expanding the design space of both the heterostructure and the metasurface.

Altogether, the present work demonstrates a substantial increase in the usable nonlinearity compared to Ref. 38 and to the existing literature. We want to highlight here that second-order nonlinear susceptibilities in the telecom spectrum increased slowly (or almost stagnated) for decades. The reviewer later brings up transition metal dichalcogenides as an example, and while these represent a promising avenue of research, they are significantly thinner than the reported material (which decreases conversion efficiency) and are incompatible with normal foundry processes (which will delay their implementation). Here, we report a dramatic increase in usable second-order nonlinearity. We therefore view this as a qualitative transition, fit for *Nature Nanotechnology*.

Comment: In particular, the authors emphasize that metasurfaces enable access to “otherwise unusable nonlinear tensor elements”; however, this capability has already been convincingly demonstrated in the nonlinear metasurface literature. For example, lithium niobate metasurfaces incorporating multiple, differently oriented metagratings have shown that the orientation of the nanostructure can directly control the polarization and nonlinear emission pathways, effectively selecting different tensor components either by patterning directly the material or in an etchless configuration [e.g. ACS Photonics 2021 8 (3), 731-737 DOI: 10.1021/acsphotonics.1c00026; Nano Lett. 23, 8091-8098 (2023) DOI: 10.1021/acs.nanolett.3c02055]. In particular, the use of GMR to enhance nonlinear optical processes is to date well established [e.g. Phys. Rev. B 106, 125411 (2022) DOI: https://doi.org/10.1103/PhysRevB.106.125411; Phys. Rev. Lett. 127, 153901 (2021) DOI: https://doi.org/10.1103/PhysRevLett.127.153901]. The authors are also among the main contributors in this field in the past decade. For instance, lithium niobate metasurfaces and related platforms have shown that careful engineering of resonant modes and polarization fields allows selective enhancement and probing of different nonlinear tensor components, as well as

efficient in- and out-coupling of light. In this context, I am afraid that the use of metasurfaces to both enhance coupling and access specific tensor elements cannot be regarded as conceptually new.

Response 2.2: We agree that metasurfaces have been widely used to enhance nonlinear optical processes and to selectively probe different tensor components, including in $LiNbO_3$ platforms. We thank the reviewer for pointing out these relevant works and have included representative references [ACS Photonics 2021; Phys. Rev. B 2022] in the revised manuscript to better contextualize our approach. We also acknowledge that the current manuscript did not sufficiently clarify how the present work differs from these established approaches, and we revised the text to make this distinction more explicit.

In prior works, GMRs were primarily used to select and enhance nonlinear processes involving co-polarized pump fields, where the relevant tensor elements can be accessed through appropriate field engineering alone. In such systems, the nonlinear overlap is not fundamentally restricted by symmetry, and the role of the metasurface is to enhance and control already accessible interactions.

In contrast, the interband MQW platform considered here involves tensor elements that require orthogonal pump field polarization components. As discussed in the manuscript, for GMRs excited at normal incidence, these field components' symmetry yields a vanishing nonlinear overlap integral. In this case, the nonlinear process is not merely weak, but symmetry-forbidden.

A key distinction of the present work is therefore that accessing these tensor elements requires not only field enhancement, but also intentional symmetry breaking of the optical mode. We revised the manuscript to highlight this advance:

> Because the resonant modes propagate along the waveguide, they can create strong E_z and E_x fields, which can in turn drive the $\chi^{(2)}_{xzx}$, $\chi^{(2)}_{xxz}$ nonlinearity. However, for GMRs excited under conventional (normal-incidence) conditions, these field components possess opposite symmetry, resulting in a vanishing nonlinear overlap integral. As a result, the corresponding nonlinear process is not simply weak, but symmetry-forbidden. Accessing these tensor elements therefore requires not only field enhancement, but also intentional symmetry breaking of the optical mode.

Our original manuscript demonstrates, through simulation and experimental verification, that the required symmetry breaking can be achieved through a small incidence angle. We now include an extended explanation in the Supplementary Information:

> **Symmetry constraints on nonlinear modal overlap**
>
> The efficiency of second harmonic generation (SHG) in the metasurface-coupled MQW system is governed by the nonlinear modal overlap between the fundamental and second harmonic fields. In the electric dipole approximation, the nonlinear polarization is given by

$$P_i(\omega) = \epsilon_0 \sum_{j,k} \chi^{(2)}_{ijk} E_j(\omega) E_k(\omega)$$

and the generated second harmonic field scales with the overlap between this nonlinear polarization and the radiating mode,

$$\beta \ \propto \ | \int_{MQW} E_{x,\omega} E_{z,\omega} E^*_{x,\omega} \, d^3x |$$

Mode symmetry at normal incidence

For GMRs excited at normal incidence, the in-plane wavevector vanishes and the excited mode is a superposition of forward and backward propagating waves with equal amplitude. The transverse field profiles are either even or odd with respect to the center of the unit cell. For TE and TM modes, the relevant field components exhibit well-defined parity. In the case of TE modes, which contain a single electric field component, this component is either even or odd. Meanwhile, TM modes contain one even and one odd component.

The nonlinear overlap integral then depends on the product of these field components. In the case of targeting the $\chi^{(2)}_{xzx}$ element, one requires a TM mode to enable the two orthogonal field components. Because E_x and E_z have opposite parity in symmetric TM modes, their product is odd, and the volume integral vanishes. As a result, the modal overlap, β, vanishes at normal incidence, and the corresponding nonlinear process is symmetry-forbidden despite the presence of non-zero local fields.

This symmetry constraint is specific to tensor elements involving orthogonal field components. For co-polarized interactions (e.g., $\chi^{(2)}_{zzz}$, the integrand remains even, and the overlap is non-zero. This explains why GMRs can efficiently enhance SHG in materials such as $LiNbO_3$, while the interband MQW tensor elements considered here remain inaccessible under symmetric excitation conditions.

Symmetry breaking

Non-zero modal overlap can be achieved by breaking the symmetry of the guided mode. This can for instance be accomplished through finite angle of incidence, or structural asymmetry of the metasurface unit cell. In both cases, the parity of the field components is modified, leading to a non-zero spatial average of the nonlinear polarization and enabling efficient SHG.

This behavior is observed in both the simulations and experiments presented in the main text, where a small angle of incidence leads to a rapid increase in the modal overlap and nonlinear signal.

Comment: Let me also add that the authors here focus more on the physics, estimating the nonlinear susceptibility. However, by looking at Figure 2f, it seems the absolute signals are not far off from the LiNbO3 thin film. How thick is the film they compared the MQW to? Looking at the numbers, I assume it is much thicker than the MQW sample.

Response 2.3: The MQW and $LiNbO_3$ thin films used for comparison have equal thicknesses (0.6 um), as mentioned in the methods. We added this information to the caption of Fig. 2:

> In both samples, the nonlinear medium is 0.6 um thick.

The observation that both samples produce comparable second harmonic flux, despite the significantly larger intrinsic nonlinear susceptibility of the MQW, highlights the importance of our work with respect to Ref. 38:

In the MQW platform, the dominant nonlinear tensor elements require a field component along the growth direction. Under free-space excitation, this component can only be introduced through oblique incidence, and even then, the large refractive index of the semiconductor limits the internal propagation angle and therefore the achievable field projection along the growth direction. As a result, the relevant nonlinear tensor elements are not efficiently accessed in the unpatterned film, and the effective nonlinear response under these conditions remains comparable to that of conventional materials such as $LiNbO_3$.

This demonstrates that intrinsic nonlinear susceptibility alone does not determine device performance in free-space configurations. By engineering the local electromagnetic fields, the metasurface enables efficient coupling to the enhanced MQW tensor elements, resulting in the large effective nonlinear response reported. This demonstrates that intrinsic nonlinear susceptibility alone does not determine device performance in free-space configurations, and that efficient utilization of these nonlinearities requires electromagnetic field engineering, as implemented here.

Comment: Here another question arises. In literature it is possible to find transition metal dichalcogenide (TMDs) materials with extremely large (at least claimed) susceptibility. Yet, given the small volumes involved the absolute conversion efficiency remains quite low before arriving to photodamage. Since this paper is clearly meant for a nanotechnology audience I believe the absolute conversion efficiency achievable in terms of P_SHG/P_in and, more crucially, conversion efficiency normalized to the input intensity should be given. I would indeed expect to see in both Figure 2f and 4b,c on the vertical axis SHG powers in W to be able to compute the conversion. From a technological point of view, I believe this is extremely relevant.

Response 2.4: In the present work, we chose to report the nonlinear response in terms of an effective $\chi^{(2)}$, extracted via comparison to a $LiNbO_3$ reference, as this provides a pump-pulse independent measure of the nonlinear interaction strength and enables the direct comparison with different material platforms.

We nevertheless agree that the conversion efficiency is useful for assessing device performance. Therefore, we calibrated our detection setup to convert our results to absolute SHG powers; however, this adds systematic uncertainty not present in the nonlinear susceptibility data. Therefore, we now include estimates of the SHG power and conversion efficiency in the Supplementary Information:

Second harmonic generation conversion efficiency

The second harmonic signal was measured using an Andor SR-500i-B2-R spectrometer with an Andor Newton 971 EMCCD detector, which records digital counts. For conversion to photon flux, we calibrated the digital count with the electron multiplication gain, quantum efficiency, and sensitivity of the detector.

The resulting photon flux was corrected for optical losses in the detection path, including mirror reflectivity, diffraction efficiency of the spectrometer and fiber efficiency. The second harmonic power was then given by $P_{SH} = N_{photons} \cdot \hbar\omega_{SH}$..

To estimate conversion efficiency, the second harmonic power was normalized to the incident pump intensity. The pump intensity was calculated from the measured average power, P_{in}, assuming a Gaussian beam profile,

$$I_{pump} = \frac{2\, P_{in}}{f_{rep}\tau A},$$

where f_{rep} is the repetition rate of the laser, τ is the pulse duration, and A is the beam area. A correction factor was applied to account for the fraction of the incident power coupled into the resonant mode, following the procedure outlined in the Methods.

Using this intensity, we define a normalized second harmonic conversion efficiency as

$$\eta_{norm} = \frac{\eta}{I_{pump}} = \frac{P_{SH}}{P_{in} \cdot\ I_{pump}}$$

We obtain a normalized second harmonic generation conversion efficiency of $\eta_{norm} \approx 1.4 \cdot 10^{-4} cm^2 GW^{-1}$, averaged over pump powers from 30 mW to 80 mW. We note that we did not observe photodamage for the powers used for this estimate. This estimate depends on assumptions regarding the beam profile, pulse duration, effective interaction area, and coupling efficiency into the resonant mode, and therefore carries a systematic uncertainty not present in the susceptibility extraction used in the main text. We note that the value should be regarded as conservative, as the experimental configuration was optimized for relative calibration of $\chi^{(2)}$against the $LiNbO_3$ reference rather than for maximizing absolute second harmonic collection efficiency.

And reference this section in the main text:

Conversion of the measured digital counts to photon number, and the procedure used to estimate absolute second harmonic powers and normalized conversion efficiencies are provided in the Supplementary Information, as their extraction relies on calibration of the detection system (including EMCCD gain, quantum efficiency, and optical collection losses) and introduces systematic uncertainties not present in the susceptibility analysis.

Comment: Something related to the maximum conversion efficiency is the possible absorption mechanisms. The change in slope in Figure 4b is attributed by the authors to a two-photon absorption process that competes with SHG. In particular, the fact that the system is based on interband SHG mechanism, where presumably resonant states are involved, could be a crucial limiting factor. I am wondering if this might result in a lower level of photodamage threshold with respect to nonlinear processes in transparent materials (between non-resonant levels).

Response 2.5: We agree that initial-state depletion effects in MQW-transition-engineered materials and two-photon absorption (TPA) in all materials can limit conversion efficiencies at high pump intensities.

Interband transitions do not suffer the initial-state depletion effects known from intersubband transitions because the initial state lies in the semiconductor's valence band. Therefore, even without doping, initial states are abundantly occupied and cannot be depleted before breaking the material: a big advantage of our material. We added a note to the manuscript:

> In contrast to intersubband MQW materials, where initial-state depletion can limit nonlinear response, interband transitions originate from the valence band and therefore do not suffer from depletion of the initial state under typical excitation conditions.

Interband transition, on-resonance, can enhance the TPA cross section (DOI:10.1063/1.349168, URL: http://purl.fcla.edu/fcla/etd/CFE0005684) by a factor of the order of ~10. Prompted by the reviewer's comment, we revisited our estimation of the light fraction lost to TPA. Our initial calculations of TPA (which are distinct from the estimation of the nonlinear susceptibility) assumed the TPA coefficient of GaAs and that the metasurface enhances the full incident power, which is spread over a large spectral band. In this case, ~1% of the incident light would be absorbed. However, due to its high quality factor, our metasurface only enhances 1.3 % of the incident femtosecond laser pulse power. Therefore, when correcting for this fact, TPA is unlikely even in the case of an MQW-enhanced TPA coefficient.

We identified the reason for the observed small deviation from quadratic scaling corrected the manuscript:

> At high powers, the second harmonic response shows a slight deviation from quadratic scaling. We attribute this to a broadening of the resonance (see Supplementary Information). We presume this stems from the spatially dependent pump beam's thermo-

optic effect, noting that only 1.3% of the pump beam's spectrum is used by the GMR, yet the full spectrum contributes thermo-optically.

The supporting figure demonstrating the quality factor scaling with pump power is included in the Supplementary Information:

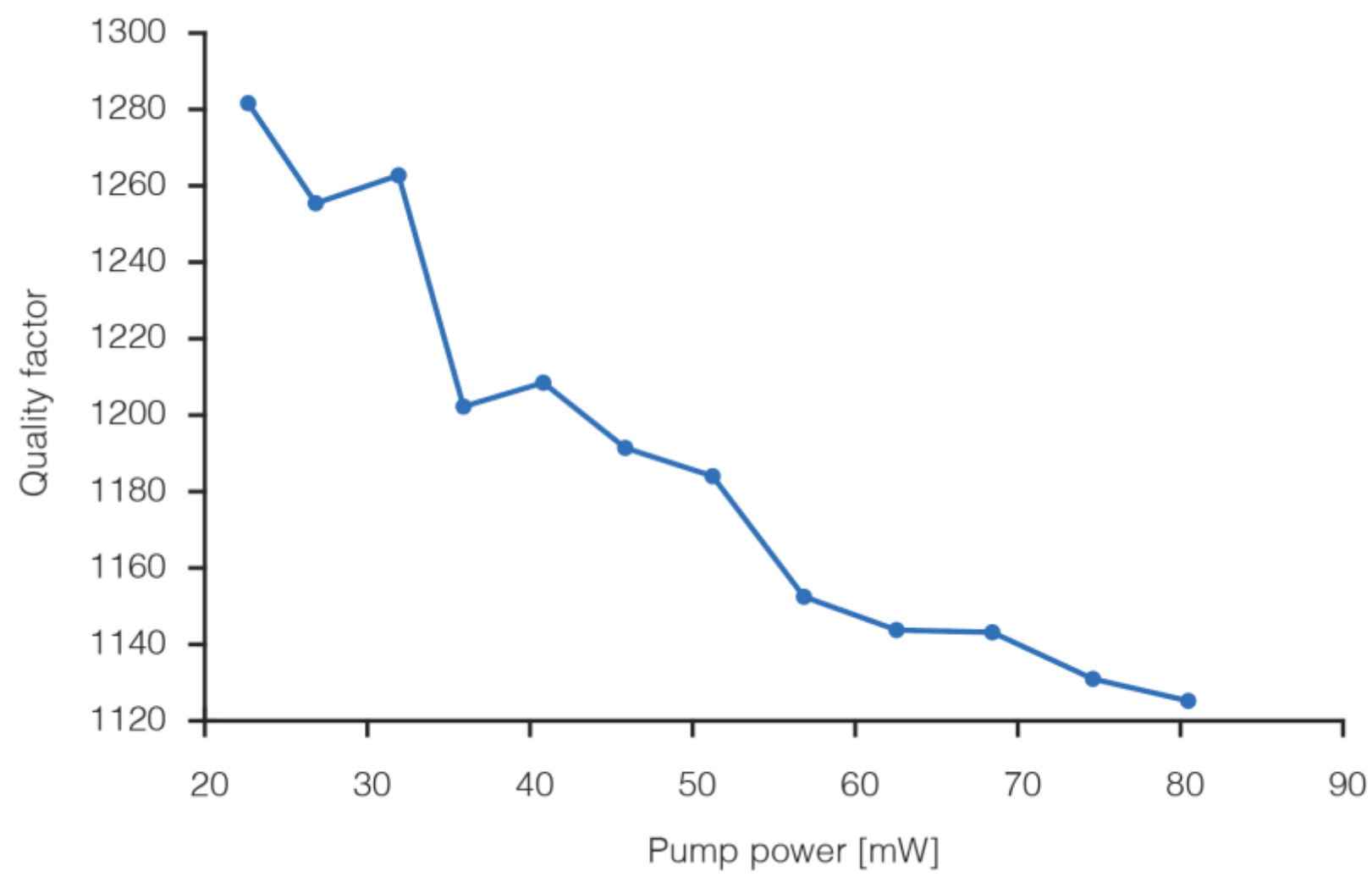

Figure R1: **Pump-power-dependent quality factor of the resonance.** The quality factor at the pump wavelength is extracted from a squared Lorentzian fit to the second harmonic response.

Even for laser intensities larger than those used in this work, interband-engineered heterostructures offer a solution: Fig. 2e shows that the second-order nonlinear susceptibility is enhanced in a broad spectral range around the exploited interband transition. If an application requires high incident powers, the MQW design can be adapted such that the transition energy exceeds twice the intended pump photon energy. This diminishes TPA but maintains a high effective $\chi^{(2)}$, enabling an optimized balance between conversion efficiency and loss. We now include this information in the manuscript:

Because the interband-enhanced nonlinear susceptibility extends over a broad spectral range, the MQW design can be adapted such that the transition energy exceeds twice the pump photon energy, thereby reducing two-photon absorption while preserving a large effective nonlinear response

Comment: Besides the above comments, which have to considered and could improve putting the paper into the right context, I believe that the primary advance of the present work lies in the photonic optimization and the performance enhancement of the structure devised in Ref [38], rather than in a fundamentally new nanotechnological concept. While the results are clearly sound and remarkable from a nonlinear optics perspective and the paper well-written, the

nanotechnological innovation—particularly in terms of heterostructure design and platform development—is limited here and was arguably more central in Ref. [38].

This distinction is crucial given the scope of Nature Nanotechnology, which emphasizes conceptual advances in nanotechnology (of course in addition to performance metrics). In this context, and considering that a group of authors—presumably those involved in the quantum well fabrication—contributed to both studies, the present manuscript reads as a natural continuation and refinement of the same platform. This raises the possibility that an integration of the two works could more clearly establish the level of conceptual advancement deserving consideration in Nature Nanotechnology. Conversely, as it stands, the current manuscript alone appears to represent an incremental step beyond prior results.

Response 2.6: We thank the reviewer for highlighting the importance of clearly positioning the conceptual contribution of this work.

We respectfully disagree with the characterization that the present manuscript represents merely a photonic optimization of the structure introduced in Ref. [38]. As stated across responses 2.1 and 2.2, the manuscript enables access to a class of nonlinear responses that are fundamentally inaccessible in conventional, symmetry-protected device geometries. It enhances their nonlinear susceptibility by a factor of 10 and, due to its quadratic scaling, this yields a ~100 times increased nonlinear response, which is a qualitative shift in a field that chases even small enhancements.

Combining the present manuscript with Ref. [38] would compromise the clarity of both works: Where Ref. [38] focuses on the heterostructure design, optimization and growth, which are of central importance to the material science community, the present manuscript experimentally demonstrates the mechanism required to make these nonlinearities usable, and demonstrates a co-design strategy for materials and nanostructures that enables tailoring all contributions to the second-order polarization. Separating the works allows a suitable focus and level of technical detail in each manuscript.

On these grounds, we maintain that the present manuscript constitutes a conceptual advance and hope the reviewer can reconsider their evaluation.

Reviewer #3 (Comments for the Author):

In this manuscript, the authors report enhanced second-harmonic generation (SHG) based on a combination of GaAs/AlGaAs multi-quantum-well (MQW) heterostructures and a metasurface supporting guided-mode resonances in the infrared. This field has been heavily studied for the past 10 years, including many publications by Igal Brener's group, a co-author of this manuscript. In this manuscript, by tuning the interband transition in the MQW heterostructures, the nonlinear coefficients $\chi_{xzx}^{(2)}$, $\chi_{xxz}^{(2)}$, $\chi_{yzy}^{(2)}$, $\chi_{yyz}^{(2)}$, were enhanced. Combined with the resonantly enhanced electric field in the z-direction induced by the guided-mode resonance, this enhancement leads to a pronounced enhancement of SHG. In addition, the manuscript presents the angular dispersion of both the linear and nonlinear responses of the designed metasurface. Although this approach is somehow new, the novelty is hardly sufficient for such a high-impact journal. This manuscript is better suited to journals such as Nano Letters or Nature Communications.

Response 3.1: Contrary to the long-established enhancement of mid-infrared nonlinearities via intersubband transitions (which were, as correctly stated, previously exploited and have been combined with plasmonic and Mie-resonant metasurfaces), our manuscript introduces an entirely new enhancement mechanism: interband transitions. This previously only theoretically predicted mechanism extends transition-engineered nonlinearities into the telecom and visible spectral ranges. Using interband transitions requires a new theoretical treatment, more careful growth, and enhances different nonlinear tensor elements. It therefore requires new metasurface designs to exploit these tensor elements. This leap is, however, worth it: we more than triple the accessible frequency range with respect to intersubband transition engineering and finally cover the telecom and visible spectrum, which are abundantly used in technology. In addition, interband transition engineering enables access to a broader range of nonlinear tensor elements than intersubband approaches, as different valence subbands (e.g., heavy-hole and light-hole bands) obey distinct selection rules. This expands the available design space for co-optimizing nonlinear materials and device architectures.

We acknowledge that the original submission did not sufficiently emphasize the conceptual novelty of the approach, and we have revised the manuscript to make this distinction more explicit. We also agree that both MQW nonlinear platforms and metasurface-enhanced second harmonic generation have been extensively studied over the past decade. However, we respectfully disagree with the assessment that the present work represents a straightforward extension of these approaches. Instead, we view it as introducing a qualitatively new class of nonlinear nanophotonic systems by enabling access to interband-engineered nonlinearities at technologically relevant wavelengths, and by rendering symmetry-forbidden nonlinear interactions accessible through tailored nanophotonic field engineering.

In previously demonstrated nonlinear guided-mode resonant metasurface platforms, including those based on $LiNbO_3$ and intersubband MQWs, the enhanced nonlinear processes rely on tensor elements that are directly accessible with co-polarized pump fields. In such systems, the

role of the metasurface is to enhance and control already allowed nonlinear interactions through field localization and mode engineering.

In contrast, the interband MQW platform considered here is governed by dominant tensor elements (e.g., $\chi^{(2)}_{xzx}$, $\chi^{(2)}_{xxz}$, $\chi^{(2)}_{yzy}$, $\chi^{(2)}_{yyz}$) that require two orthogonal electric field components at the fundamental frequency. For GMRs under conventional excitation, these field components possess opposite symmetry, leading to a vanishing nonlinear overlap integral. As a result, the corresponding nonlinear process is not simply weak, but symmetry-forbidden for guided modes. Even when the required field components exist within the structure, their symmetry prevents constructive nonlinear coupling, resulting in zero net second harmonic generation.

The key conceptual advance of this work is the demonstration that such symmetry-protected nonlinear interactions can be unlocked through intentional symmetry breaking of the electromagnetic mode. We show both theoretically and experimentally that by combining bandstructure-engineered MQWs with guided-mode-resonant metasurfaces, it is possible to simultaneously (i) generate the required field components and (ii) break the symmetry that otherwise suppresses the nonlinear overlap. This enables access to a class of nonlinear tensor elements that have not been previously realized in nanophotonic devices.

We agree that this distinction was not made sufficiently explicit in the original manuscript. To address this, we have revised the text to clearly highlight the role of symmetry protection and its breaking as the central physical mechanism of the work. In particular, we have added the following discussion:

> Because the resonant modes propagate along the waveguide, they can create strong E_x and E_z fields, which can in turn drive the $\chi^{(2)}_{xzx}$, $\chi^{(2)}_{xxz}$ nonlinearity. However, for GMRs excited under conventional (normal-incidence) conditions, these field components possess opposite symmetry, resulting in a vanishing nonlinear overlap integral. As a result, the corresponding nonlinear process is not simply weak, but symmetry-forbidden. Accessing these tensor elements therefore requires not only field enhancement, but also intentional symmetry breaking of the optical mode.

From this perspective, the present work does not represent a quantitative improvement within an established framework, but rather a qualitative extension of nonlinear metasurface concepts into a regime where both the nonlinear material response and its optical accessibility must be co-engineered. By combining interband band structure design with symmetry-engineered nanophotonic modes, we move beyond enhancing existing nonlinear processes to enabling interactions that are otherwise inaccessible in conventional platforms. Rather than relying on naturally available nonlinearities, the approach demonstrated here enables both the magnitude and accessibility of the nonlinear response to be engineered simultaneously. We believe this dual advance constitutes a conceptual step aligned with the scope of *Nature Nanotechnology*.

Comment: Nevertheless, my detailed comments and concerns are outlined below.

The authors use an x-cut LiNbO3 thin film as a reference to demonstrate the nonlinearity of the reported MQW heterostructures. Although LiNbO3 may exhibit a comparable χ_zzz^((2)) coefficient, its second-order nonlinear tensor is different from that of GaAs/AlGaAs. Other tensor components may also contribute to the observed response. Why did the authors not use a GaAs/AlGaAs thin film as a more appropriate reference for comparison?

Response 3.2: We agree that the exploited nonlinear tensor element of $LiNbO_3$ differs from that of the MQW. However, in the MQW system, the dominant nonlinear tensor elements arise from band structure engineering rather than directly from the intrinsic tensor of the constituent materials. Due to selection rules, the interband-enhanced second-harmonic nonlinear susceptibility tensor elements also require different light polarizations than bulk GaAs and AlGaAs. As a result, the effective nonlinear response of the MQW cannot be mapped one-to-one onto that of any bulk reference material, including GaAs or AlGaAs.

Importantly, we explicitly account for the field polarization in, and tensor structure of each material. In particular, the electric field components within each structure are computed, and the resulting nonlinear polarization is evaluated using the appropriate tensor elements for that material. $\chi^{(2)}$ was then extracted by matching simulated and measured second harmonic signals. This approach ensures that differences in tensor symmetry between $LiNbO_3$ and the MQW are accounted for.

Our key requirement for the reference material was thus not (conventionally unattainable) tensor equivalence, but a reliable nonlinear response. $LiNbO_3$ is particularly well-suited in this regard: it is (1) non-absorbing at second harmonic wavelengths, (2) an extensively studied and abundantly applied nonlinear material, and (3) high-quality thin-film samples with reproducible properties are commercially available. We thus deem it the most expressive benchmark for the MQW's nonlinear response. We include this in the main text:

> $LiNbO_3$ is chosen as a well-characterized, low-loss nonlinear reference material with reproducible thin-film properties, enabling a reliable and well-calibrated benchmark for the MQW nonlinear response.

Regarding the reviewer's concern that other tensor components may contribute to the measured signal, we note that the use of polarization-resolved excitation and detection allows us to isolate the relevant nonlinear response. In the case of $LiNbO_3$, this corresponds to $\chi^{(2)}_{zzz}$, while for the MQW, we extract the sum of $\chi^{(2)}_{xzx} + \chi^{(2)}_{xxz}$, which are fundamentally indistinguishable in degenerate second harmonic generation.

Comment: The refractive index is a crucial parameter in the design of guided-mode resonances, particularly for predicting their Q-factors and angular dispersion. Could the authors provide the effective refractive indices of the MQW heterostructures and TiO2?

Response 3.3: The refractive index of the MQW heterostructure was measured using spectroscopic ellipsometry and is provided in the Supplementary Information (Fig. S1b).

Following the reviewer's suggestion, we also included the measured refractive index of the TiO_2 thin film used in the metasurface fabrication. This was obtained via spectroscopic ellipsometry on an amorphous TiO_2 layer deposited by atomic layer deposition under the same conditions as the metasurface. These experimentally measured refractive indices were used directly in the numerical simulations presented in the manuscript.

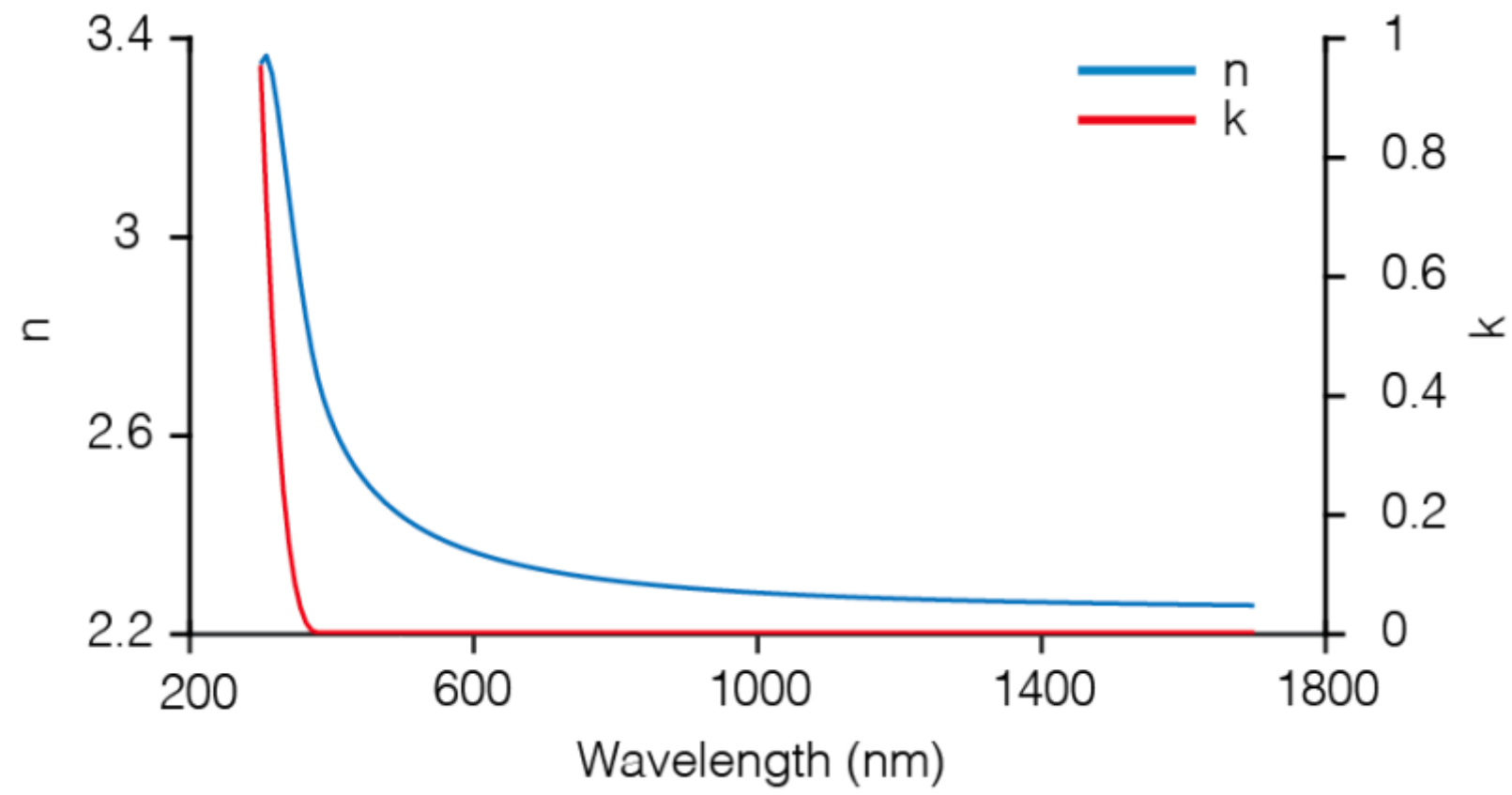

Figure R2: **Characterization of TiO_2.** Refractive index of an atomic-layer-deposited TiO_2 thin film on a Si substrate, measured by spectroscopic ellipsometry (Woollam RC2).

Comment: In Fig. 3(b), only the electric-field distributions in the xy plane are presented. Since the field enhancement associated with guided-mode resonances is typically non-uniform across the thickness of the film, especially within the MQW heterostructures, could the authors also provide the electric-field distributions in the xz or yz plane? Could the authors clarify how the field distribution across the thickness of the film would affect the SHG enhancement?

Response 3.4: We added the electric field distributions of the yz plane to the Supplementary Information:

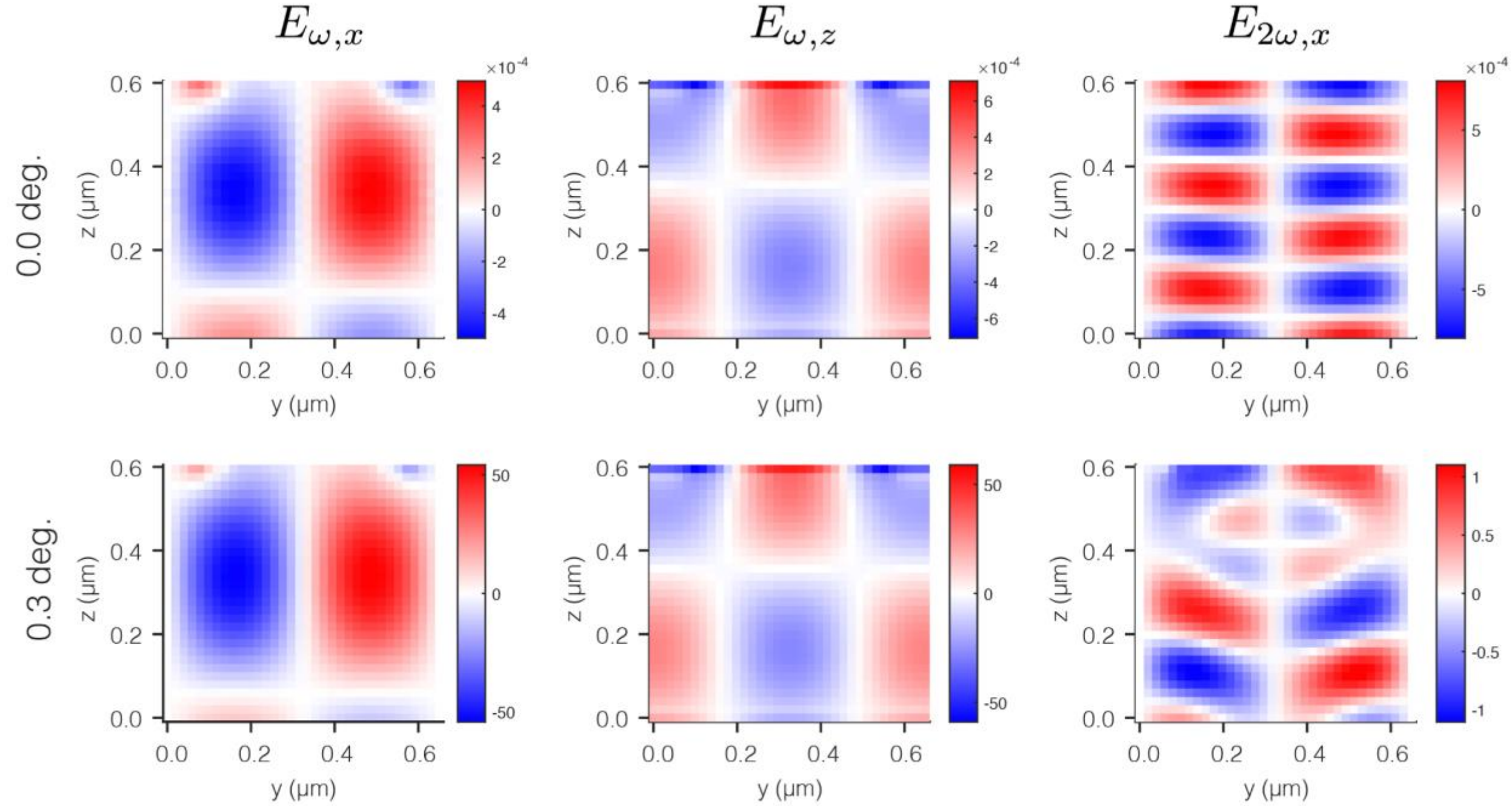

Figure R3: **Field distribution in the waveguide at resonance.** The panels show the spatial (yz) profile of the simulated fields (arb. units) in the waveguide, summed along the x-direction of the unit cell. We show the x and z component of the pump field and the x component of the second harmonic field for normal and 0.3° incidence angle for the left branch of resonance B.

The results confirm that the GMRs produce non-uniform field distributions across the thickness of the structure. The impact of this non-uniformity on SHG is captured by the modal overlap integral, which depends on the spatial overlap of the fundamental and second harmonic fields within the MQW layer.

Importantly, the nonlinear response is not dominated by localized hotspots. While the field distribution is non-uniform, it remains extended throughout the nonlinear medium, such that the SHG enhancement arises from the distributed overlap of the relevant field components across the MQW region. This extended interaction volume enables efficient nonlinear conversion while avoiding excessive localization that could increase nonlinear absorption in confined regions.

Comment: In Fig. 3(f), the overlap factor (β) appears to remain very small for resonance C, suggesting that the electric field in the z-direction is not enhanced. Could the authors clarify the reason for this?

Response 3.5: The small overlap factor of resonance C arises from its field polarization. Its field components are largely confined to in-plane components, with only a weak out-of-plane component. Since the dominant tensor element of the MQW film requires orthogonal pump photon

polarizations (one in-plane and one out-of-plane polarized photon), this leads to a suppressed overlap factor and weak second harmonic generation contribution from this resonance.

This is consistent with the observed angular behavior discussed in the manuscript: unlike resonances A and B, resonance C does not split under rotation about the y-axis, indicating that this resonance couples to guided modes with momentum oriented along the orthogonal lattice orientation. Rotation about the x-axis enables splitting of resonance C. Here, the resonance couples to modes with both in- and out-of-plane electric field components, enabling large β. This demonstrates that the weak response observed in Fig. 3(f) is not an intrinsic limitation of the resonance, but a consequence of the specific excitation geometry.

The figure caption for Fig. 3(f) now includes:

> The electric field of resonance C is largely confined to in-plane components, thereby suppressing the modal overlap compared to resonances A and B.

Comment: Why do the overlap factor (β) and the SHG intensity both reach their maximum at an incident angle of 0.3°? Is this primarily related to the Q-factor, the field distribution, or another mechanism? A clear and detailed explanation is needed, particularly given the substantial variation in Q-factor. At present, it is still unclear why the SHG emission differs so markedly between incident angles of 0.3° and 1°.

Response 3.6: We agree that this point should be more explicitly addressed in the manuscript, and the manuscript now reads:

> The largest second harmonic response is observed at small incidence angles. This is a result of two contributing factors: (1) the resonance Q-factor decreases with increasing angle, reducing field enhancement, and (2) at small angles, the two angle-split GMR branches remain spectrally overlapped, contributing additively to the second harmonic signal. At larger angles, the branches separate and contribute independently, reducing the combined response.

We note that the device was designed to operate at a small, non-zero angle of incidence, while the specific maximum observed at 0.3° arises from the experimental realization of the resonance. This maximum does not reflect a fundamental optimum of the nonlinear interaction, but results from the combination of a high quality factor at this angle and the spectral overlap of the two resonance branches.

Comment: Since the in-plane symmetry is broken when the incident angle is varied, the authors should specify the direction along which the incident angle is tuned, that is, whether the change is introduced through the px or py momentum component.

Response 3.7: We now include an illustration of the rotation angle in Fig. 3a and specify the rotation axis and momentum component in the figure caption.

The sample is rotated about the y-axis, introducing a non-zero k_x momentum component

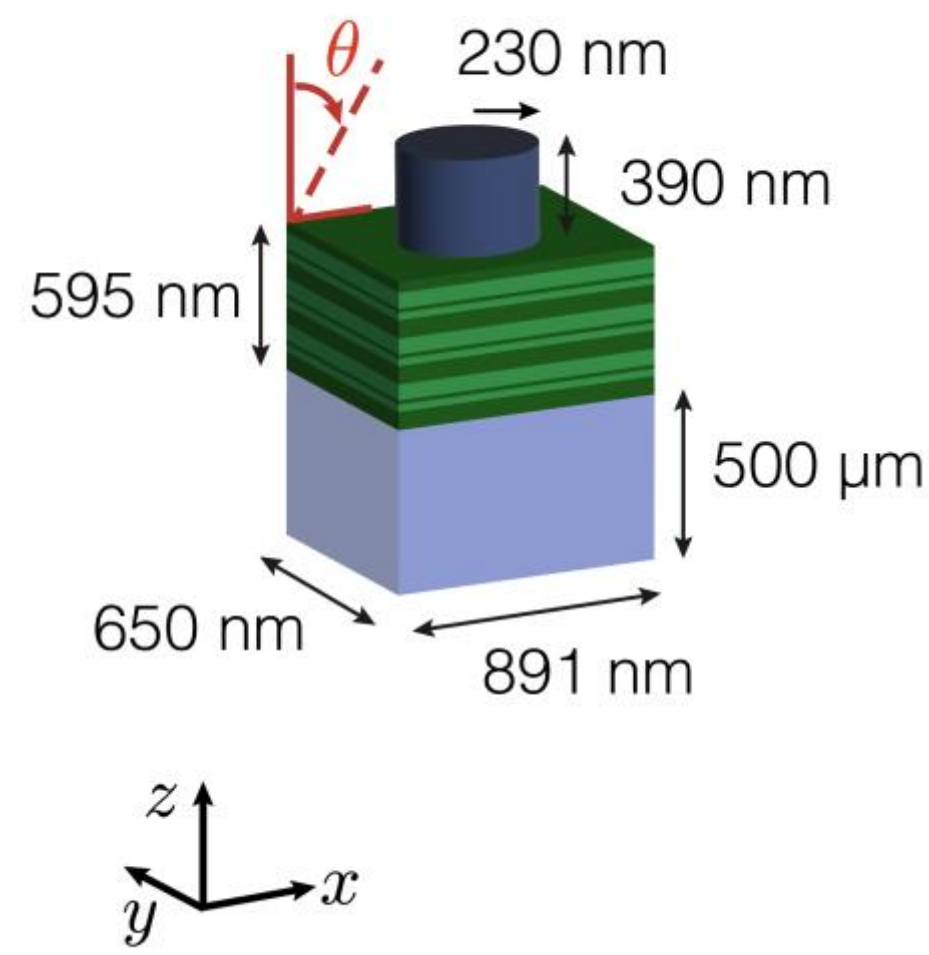

Figure R4: Metasurface unit cell with dimensions (dark blue: TiO_2, green: heterostructure, light blue: Al_2O_3). The sample is rotated about the y-axis, introducing a non-zero k_x momentum component.
